# RGS3 acts as a tumor promoter by facilitating the regulation of the TGF-β signaling pathway and promoting EMT in ovarian cancer

**DOI:** 10.1038/s41420-025-02536-3

**Published:** 2025-06-02

**Authors:** Zizhao Wang, Huating Sun, Shunpeng Zhu, Fang Wang, Quan Li, Jinhua Zhou

**Affiliations:** 1 Department of Obstetrics and Gynecology, The First Affiliated Hospital of Soochow University, Suzhou, China; 2 Clinical Research Center of Obstetrics and Gynecology, Jiangsu Key Laboratory of Clinical Immunology of Soochow University, Suzhou, China; 3 Jiangsu Institute of Clinical Immunology, The First Affiliated Hospital of Soochow University, Suzhou, China

**Keywords:** Predictive markers, Tumour biomarkers

## Abstract

Ovarian cancer (OC) is one of the most common and lethal solid malignancies among women, with its incidence steadily rising. Despite substantial advancements in OC research, its pathogenesis remains largely elusive. Recent studies indicate that the regulator of G protein signaling 3 (RGS3) is implicated in tumorigenesis, however, its specific role in OC development has not been extensively investigated. Herein, this research elucidated that the overexpression of RGS3 in OC correlates with adverse clinical pathological features and tumor progression. Furthermore, we demonstrated that silencing RGS3 promotes apoptosis, effectively inhibiting tumor growth and metastasis. Additionally, our findings reveal that RGS3 enhances oncogenic activity by participating in the regulation of the transforming growth factor-beta (TGF-β) pathway and corresponding epithelial-mesenchymal transition (EMT). The in-depth mechanism lies in the RGS3 facilitating the phosphorylation of SMAD2/3 by directly interacting with AT-rich interactive domain-containing protein 3B (ARID3B), which ultimately drives OC cell proliferation and metastasis. Therefore, our results position RGS3 as a significant prognostic biomarker and tumor-promoting factor in OC, underscoring the pivotal role of the RGS3/TGF-β/EMT signaling pathway in the pathogenesis of this malignancy.

## Introduction

Ovarian cancer (OC) is one of the most prevalent malignancies of the female reproductive system and has the highest mortality rate among gynecologic cancers [1], with China reporting the highest incidence in 2022 [2]. Despite advancements in chemotherapy, radiotherapy, and targeted therapies for OC, most cases are diagnosed at advanced stages due to its unclear etiology and the lack of effective early detection methods, characterized by extensive metastasis [3, 4]. Therefore, identifying early biomarkers and potential therapeutic targets is essential for improving clinical outcomes.

G protein-coupled receptors (GPCRs) play a crucial role in maintaining cellular homeostasis and are well-established drug targets, given their significant involvement in tumor progression as key mediators of cellular signaling [5]. The regulator of G protein signaling (RGS) family, a diverse group of multifunctional proteins, participates in the intracellular signaling of GPCRs, with several members implicated in the pathogenesis of various cancers [6]. Recent research has increasingly focused on the role of the RGS gene family in OC [7, 8].

Notably, a five-gene risk score comprising RGS11, RGS10, RGS13, RGS4, and RGS3 has been identified, establishing this score as an independent prognostic marker for OC [9]. RGS3 was found to be significantly upregulated in OC among these. However, no experimental evidence has yet confirmed the role of RGS3 in OC. RGS3, a cytoplasmic protein-coding gene, is known for its overexpression in various malignancies and its role in oncogenesis. It inhibits G-protein-mediated signaling by enhancing the GTPase activity of G-protein alpha subunits, thereby downregulating heterotrimeric G-protein signaling [10]. Emerging oncological evidence suggests that RGS3 may contribute to the progression of tumor metastasis of various cancers, including gastric [11], liver [12], and lung cancers [13]. In OC, the proliferation and migration phenotypes are particularly critical for tumor metastasis, yet RGS3’s role remains underexplored.

In this study, we demonstrated that RGS3 interacts with ARID3B to promote the phosphorylation of SMAD2/3, participating in the regulation of the transforming growth factor β (TGF-β) signaling pathway and promoting the malignant traits of OC cells. This reveals a novel aspect of RGS3’s role in cancer biology, offering potential new avenues for therapeutic intervention.

The TGF-β signaling pathway regulates various cellular processes, including proliferation, migration, apoptosis, differentiation, as well as tumor initiation and progression [14]. SMAD proteins are key transducers in the TGF-β pathway, transmitting extracellular signals directly to the nucleus. Activation of the TGF-β signaling cascade begins when TGF-β binds to its transmembrane receptors, TGF-βRI and TGF-βRII, triggering SMAD2/3 phosphorylation. While the role of TGF-β/SMAD signaling in tumorigenesis can be oncogenic, as tumors progress, cancer cells often evade suppressive signals and begin secreting TGF-β proteins, which promote angiogenesis, metastasis, and immune evasion. Moreover, TGF-β-induced EMT is a key factor in tumor invasiveness and metastasis [15].

Herein, we are the first to demonstrate that RGS3 is overexpressed in serous OC tissues, lymph node metastases, and OC cell lines, with significantly elevated levels in metastatic lesions. RGS3 appears to drive OC progression by regulating the TGF-β signaling pathway and inducing EMT, functioning as a major oncogenic driver. Importantly, we identified ARID3B as a key mediator in RGS3-induced TGF-β regulation. RGS3 directly interacts with ARID3B to facilitate SMAD2/3 phosphorylation, amplifying TGF-β signaling through its interactions. Our findings highlight the critical role of RGS3 in the TGF-β signaling pathway during tumorigenesis, positioning RGS3 as a potential therapeutic biomarker for OC.

## Results

### RGS3 is up-regulated in OC and acts as a tumor promoter in clinical samples

**Fig. 1:**
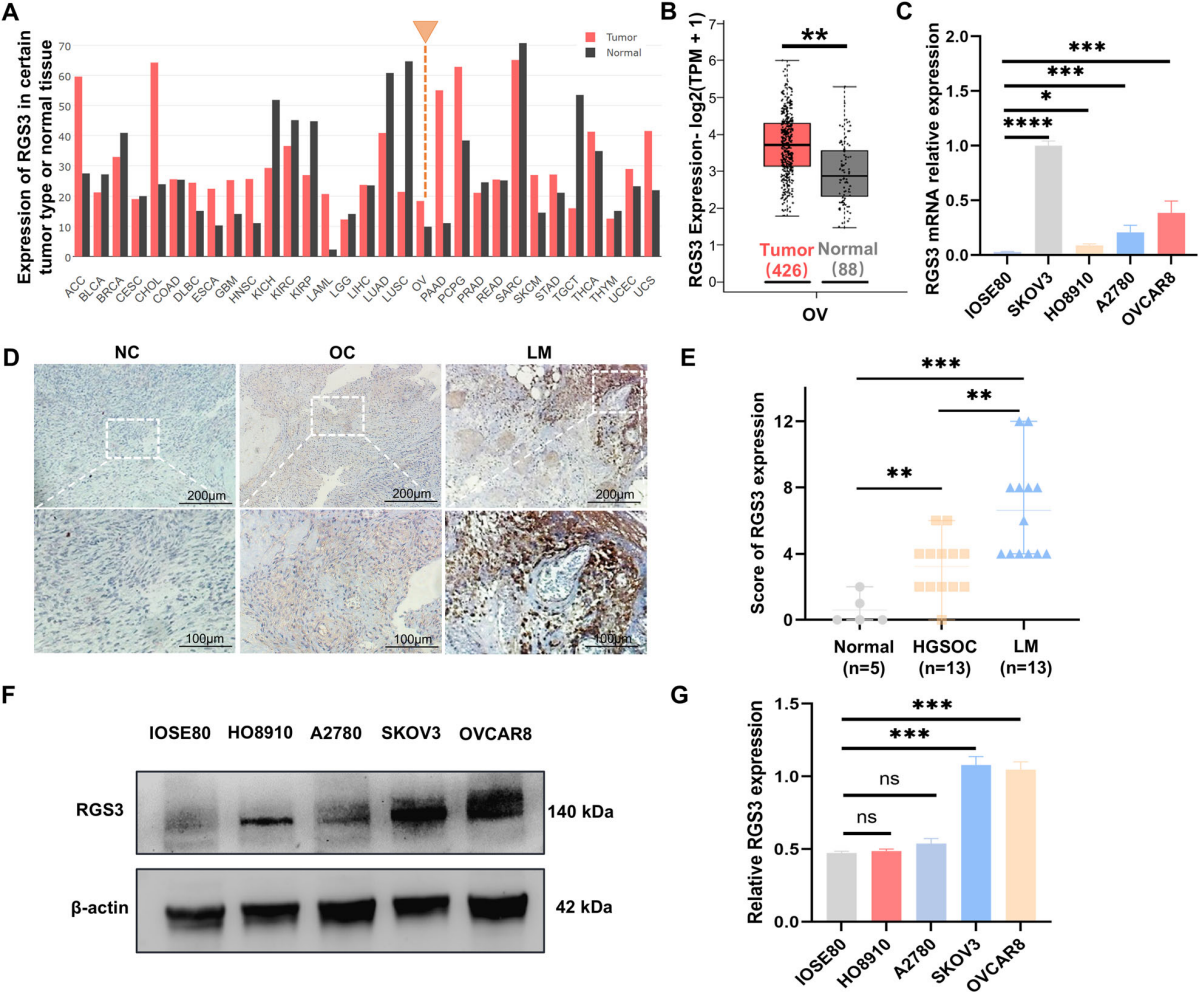
RGS3 upregulation in OC tissues and cell lines. **A** GEPIA database showing high RGS3 expression across multiple tumors. **B** RGS3 expression levels are significantly higher in ovarian serous carcinoma compared to normal ovarian tissues in the GEPIA database. **C** RT-qPCR results demonstrating increased RGS3 expression in O cell lines. **D** Representative IHC images showing RGS3 expression in normal ovarian tissues (NC), ovarian carcinoma tissues (OC), and lymph node metastases (LM). **E** IHC scoring indicates higher expression in metastatic compared to primary tumors. **F**, **G** Western blot analysis showing elevated RGS3 in various OC cell lines. Data were analyzed using Student’s *t*-test. Data represent mean ± SD of three independent experiments. ^*^P < 0.05, ^**^P < 0.01, ^***^P < 0.001, ^****^P < 0.0001, (scale bars: 200 μm, 100 μm).

Initially, we conducted differential analysis using public databases to investigate the role of RGS3 in tumorigenesis. Our study identified significant overexpression of RGS3 in OC tissues and cell lines. Analysis from the GEPIA database analysis revealed that RGS3 is upregulated in multiple tumor types, with notably higher mRNA levels in ovarian serous carcinoma compared to normal ovarian surface epithelium (Fig. 1A, B). RT-qPCR further confirmed the elevated RGS3 expression in various OC cell lines (Fig. 1C). Additionally, we conducted IHC experiments on 5 normal ovarian tissues, 13 primary tumor sites, and 13 lymph node metastases, with representative images displayed in Fig. 1D. Both primary and metastatic OC tissues exhibited significantly higher IHC scores compared to normal tissues, with the highest scores observed in lymph node metastases (Fig. 1E). However, RGS3 expression did not correlate with age and TNM stage (Table S1). Western blot analysis supported these findings, showing increased RGS3 expression in most OC cell lines (Fig. 1F, G). Collectively, these results suggest that RGS3 plays a crucial role in the progression of OC.

### RGS3 deficiency inhibits OC cell proliferation and metastasis in vitro

**Fig. 2:**
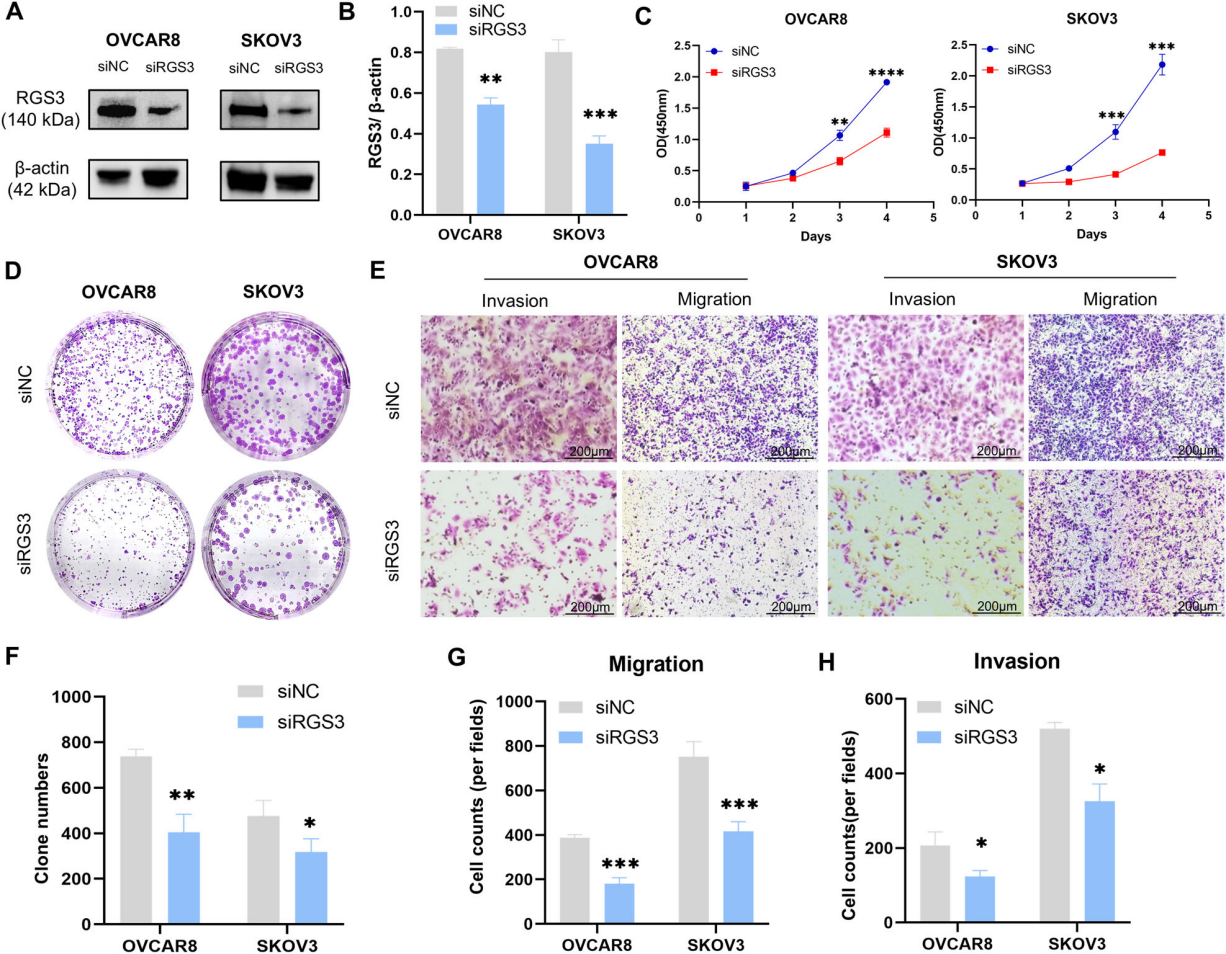
RGS3 deficiency inhibits OC proliferation and metastasis in vitro. **A**, **B** Western blot confirmation of siRGS3 transfection efficiency. **C** CCK8 assay measuring cell viability in SKOV3 and OVCAR8 cells post-siRGS3 transfection. **D**, **F** Colony formation assay images and quantification post-siRGS3 transfection. **E**, **G**, and **H** Transwell assays showing reduced migration and invasion in siRGS3-transfected cells. Data represent mean ± SD of three independent experiments. Differences between the two groups were analyzed using Student’s *t*-test. ^*^P < 0.05, ^**^P < 0.01, ^***^P < 0.001, (scale bar *=* 200 μm).

Our findings indicate that RGS3 deficiency suppresses OC cell proliferation both in vitro and in vivo, which is critical for tumorigenicity. To examine the role of RGS3 in tumor growth, SKOV3 and OVCAR8 cells were transfected with siRGS3, and Western blotting confirmed siRNA transfection efficiency (Fig. 2A, B). We assessed the effects on cell viability and proliferation using CCK8 and colony formation assays. The CCK8 assay revealed that RGS3 knockdown significantly inhibited cell viability from 0 to 96 h post-transfection compared to controls (Fig. 2C), and colony formation was markedly reduced (Fig. 2D, F). These results suggest that silencing RGS3 inhibits the proliferative activity of SKOV3 and OVCAR8 cells.

To assess RGS3’s impact on metastasis, we performed Transwell migration and invasion assays. SKOV3 and OVCAR8 cells transfected with siRGS3 exhibited significantly reduced migration and invasion compared to control siRNA-transfected cells (Fig. 2E, G, H).

### RGS3 overexpression enhances OC cell proliferation and metastasis in vitro

To further investigate the impact of RGS3 on OC cell proliferation and metastasis, we overexpressed RGS3 in SKOV3 and OVCAR8 cells via plasmid transfection, confirmed through Western blotting (Fig. S1A, B). CCK8 assays revealed that RGS3 overexpression significantly enhanced cell viability from 0 to 96 h post-transfection compared to the vector group (Fig. S1C), while colony formation assays showed a marked increase in colony number (Fig. S1D, F). Transwell assays revealed that RGS3 overexpression significantly increased cell migration and invasion capabilities in both cell lines (Fig. S1E, G, and H).

### RGS3 inhibits the apoptosis of OC cells

**Fig. 3:**
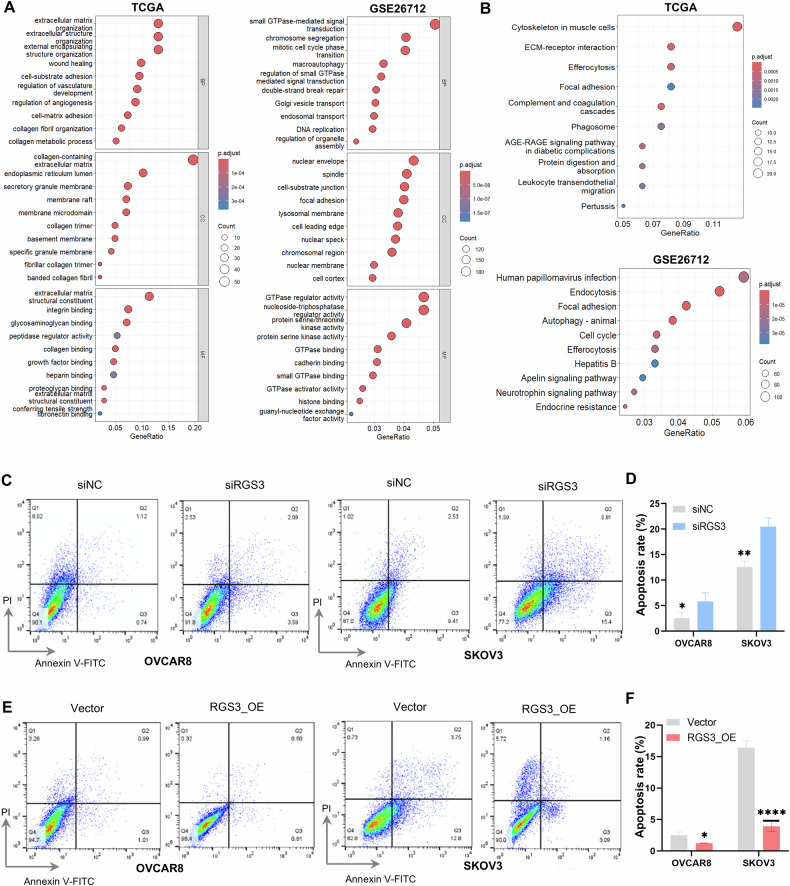
RGS3 inhibits apoptosis of OC cells. **A** Gene Ontology enrichment analysis of RGS3 based on TCGA and GSE26712 databases. **B** Kyoto Encyclopedia of Genes and Genomes pathway analysis of RGS3 based on TCGA and GSE26712 database. **C**–**F** Apoptosis rates were measured with Annexin V FITC/PI staining. Data represent mean ± SD of three experiments. Differences between the two groups were analyzed using Student’s *t*-test. ^*^P < 0.05*,* ^**^P < 0.01, ^***^P < 0.001*,* ^****^P < 0.0001.

To further investigate the physiological function of RGS3, we utilized the rcorr algorithm from the Hmisc package in R to identify RGS3-associated co-expressed genes (*R* > 0.3, *P* < 0.001) in the TCGA and GSE26712 datasets. GO analysis revealed that RGS3 is likely localized to the cytoplasm and cell membrane, and is involved in processes such as protein catabolism, cell growth, apoptosis, and integrin signaling (Fig. 3A). KEGG analysis indicated that RGS3 and its co-expressed genes may regulate ECM-receptor interactions, focal adhesion signaling, and amino acid biosynthesis pathways (Fig. 3B). Based on these insights, we employed flow cytometry to assess the specific role of RGS3 in apoptosis. Apoptosis assays showed that RGS3 silencing increased the proportion of apoptotic cells in both cell lines (Fig. 3C, D). Conversely, overexpression of RGS3 reduced apoptosis in OVCAR8 and SKOV3 cells (Fig. 3E, F). Collectively, these findings suggest that RGS3 is involved in the apoptosis of OC cells, underscoring its potential significance in cancer biology.

### RGS3 knockdown suppresses tumor proliferation in vivo

**Fig. 4:**
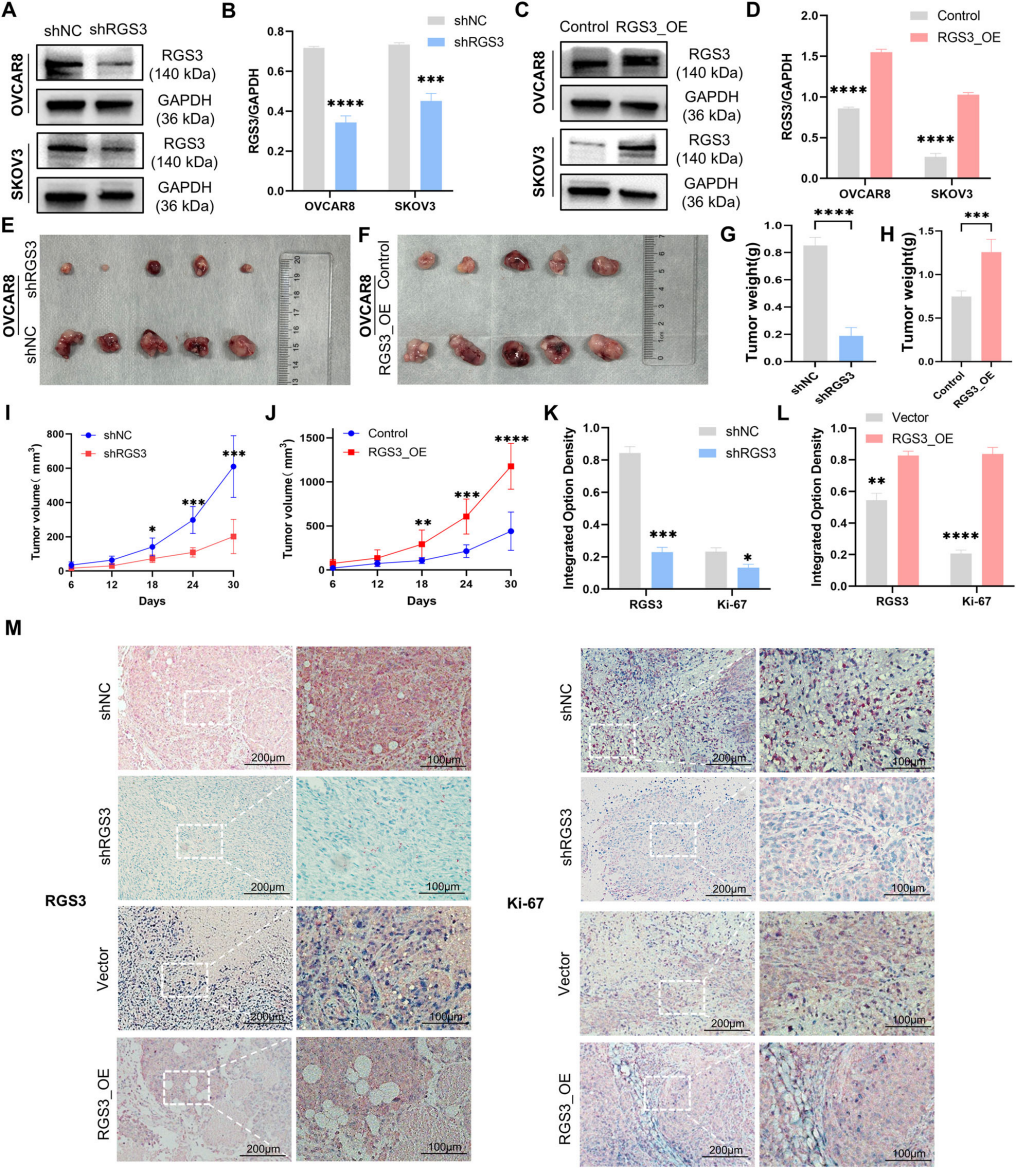
RGS3 knockdown inhibits tumor proliferation in vivo. **A**–**D** Western blot analysis of infection efficiency in SKOV3 and OVCAR8 cells with lentiviral constructs. **E**, **F** Xenograft tumor growth and size comparison between RGS3 knockdown and overexpression groups. **G**–**J** Xenograft tumor volume and weight comparison between RGS3 knockdown and overexpression groups. **K**, **L** The expression of Ki-67 and RGS3 was measured in IOD. **M** Representative immunohistochemical images of RGS3 and Ki-67 expression. Data analyzed using Student’s *t*-test, ^*^P < 0.05, ^**^P < 0.01, ^***^P < 0.001, ^****^P < 0.0001, (scale bars: 200 μm, 100 μm).

To evaluate the effect of RGS3 on tumor growth in vivo, we infected SKOV3 and OVCAR8 cells with lentiviruses expressing either shRGS3 or control constructs. Western blot confirmed the infection efficiency (Fig. 4A–D). In a Nod-SCID mouse xenograft model, at the end of the study, RGS3 knockdown significantly slowed tumor growth and reduced tumor size compared to the control group, while RGS3 overexpression accelerated tumor growth and increased tumor size (Figs. 4E, F and S1I). Tumor volume and weight were notably reduced in the knockdown group, and increased in the overexpression group (Fig. 4G, H, I, J). Immunohistochemical analysis revealed that RGS3 and Ki-67 expression levels were lower in tumors with RGS3 knockdown, while they were higher in tumors with RGS3 overexpression (Fig. 4M). The integrated option density was used to analyze the proportion of positive signals in the IHC images (Fig. 4K, L).

### Knockdown of RGS3 inhibits tumor metastasis potential in vivo

**Fig. 5:**
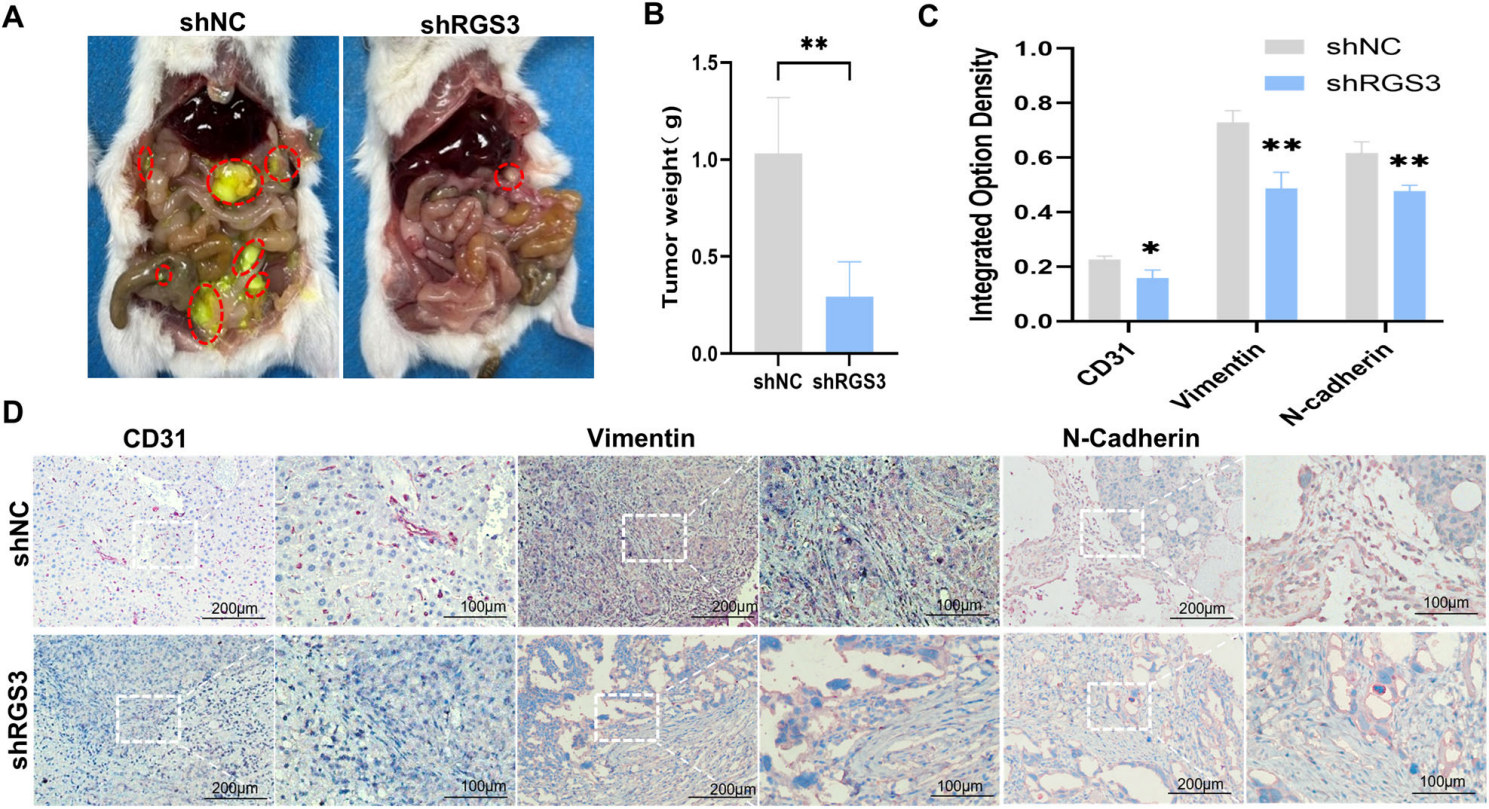
RGS3 knockdown reduces metastasis potential in vivo. **A**, **B** Tumor morphology and weight assessment 7 weeks after intraperitoneal injection of shRGS3 and control OVCAR8 cells. **C** The expression of RGS3, CD31, vimentin, and N-Cadherin was measured in IOD. **D** IHC analysis of RGS3, CD31, vimentin, and N-Cadherin expression in RGS3-knockdown tumors. ^*^P < 0.05, ^**^P < 0.01, ^***^P < 0.001, ^****^P < 0.0001, (scale bars: 200 μm, 100 μm).

To assess the impact of RGS3 knockdown on metastasis, we established a peritoneal injection metastasis model using OVCAR8 cells infected with shRGS3 or control lentiviruses in Nod-SCID mice. Control cells formed multiple visible metastatic tumors, leading to significant ascites and increased tumor number and size within the peritoneal cavity. In contrast, RGS3-knockdown cells formed fewer metastatic lesions, with a significant reduction in tumor weight (Fig. 5A, B). IHC analysis of the tumor tissues revealed decreased expression of CD31, Vimentin, and N-Cadherin in RGS3-knockdown tumors (Fig. 5C, D). These findings demonstrate that RGS3 knockdown inhibits OC metastasis in vivo, highlighting its potential as a therapeutic target for OC.

### Knockdown of RGS3 suppresses tumor progression via the TGF-β signaling pathway and EMT

**Fig. 6:**
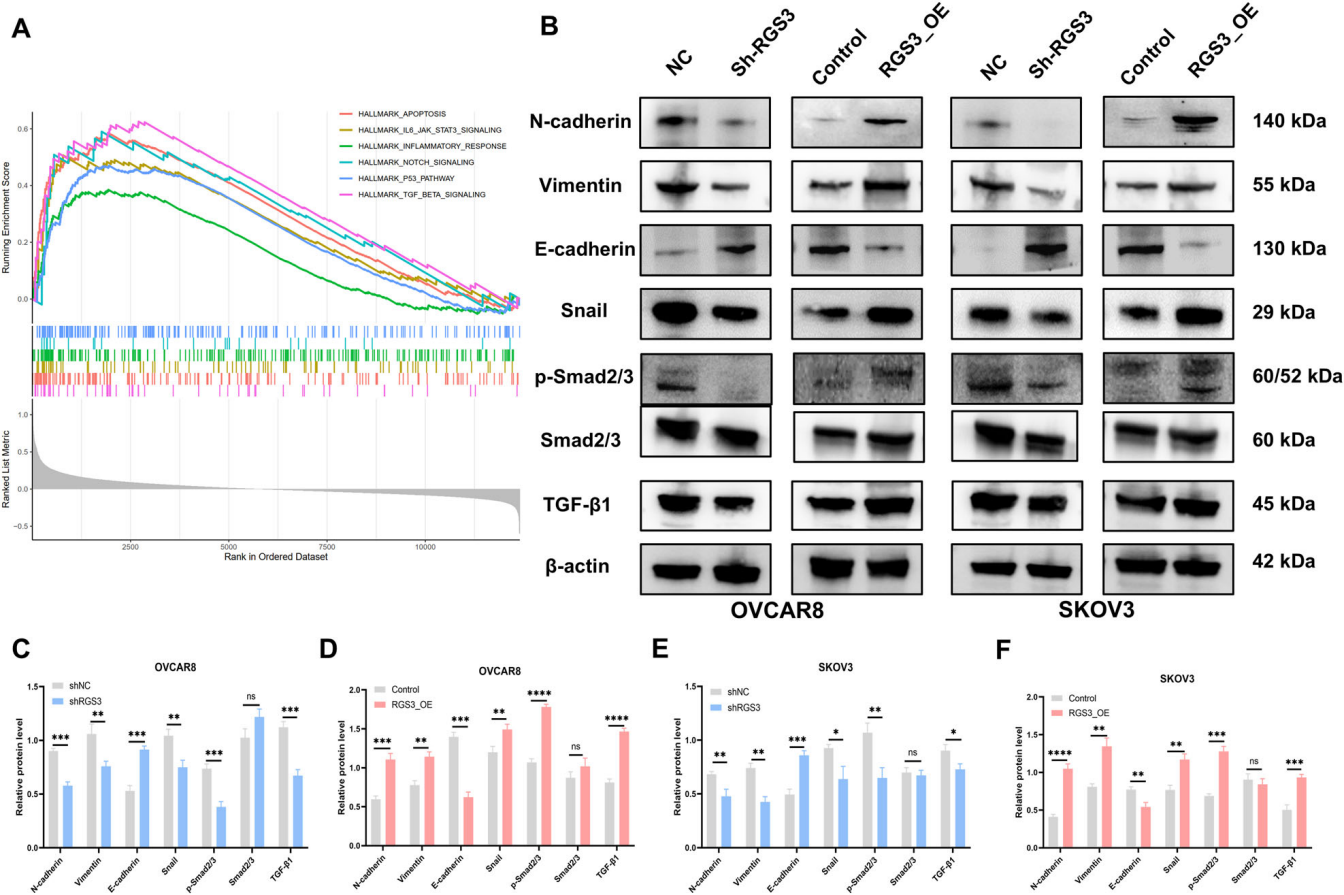
RGS3 promotes tumor progression via the TGF-β signaling pathway and EMT. **A** Gene set enrichment analysis of bioinformatic analysis of high and low RGS3 expression groups in GSE26712. **B** Effects of RGS3 knockdown and overexpression on TGF-β/EMT markers in OVCAR8 and SKOV3 cells. **C**–**F** Quantification of TGF-β/EMT protein expression using ImageJ software. NS not significant, ^*^P < 0.05, ^**^P < 0.01, ^***^P < 0.001, ^****^P < 0.0001.

To further explore the downstream pathways and molecules, we ranked RGS3 expression levels in the GSE26712 dataset from high to low, using the median as the cutoff to define high and low RGS3 expression groups (|Log2Fold Change| > 0.5). GSEA was then conducted to identify pathways differentially activated between the high and low RGS3 expression groups. As shown in Fig. 6A, we observed significant enrichment in pathways such as JAK-STAT3(Normalized Enrichment Scores (NES) = 1.73), NOTCH (NES = 1.69), P53 (NES = 1.76), and notably, the TGF-β signaling pathway (NES = 1.98).

Given that the TGF-β pathway is a central driver of EMT [16], we further investigated the role of RGS3 in this pathway by assessing the expression of key TGF-β and EMT-related molecules in SKOV3 and OVCAR8 cells following RGS3 knockdown or overexpression. Western blot analysis revealed that RGS3 knockdown significantly reduced the phosphorylation of Smad2/3 without altering total Smad2/3 protein levels. Additionally, RGS3 knockdown decreased the expression of TGF-β1, N-cadherin, vimentin, and Snail, while increasing E-cadherin levels. In contrast, RGS3 overexpression had the opposite effect (Fig. 6B–E). These results suggest that RGS3 promotes OC cell proliferation and metastasis by participating in the regulation of the TGF-β pathway and inducing EMT.

### Identifying the downstream effectors of RGS3 in TGF-β signaling pathway

**Fig. 7:**
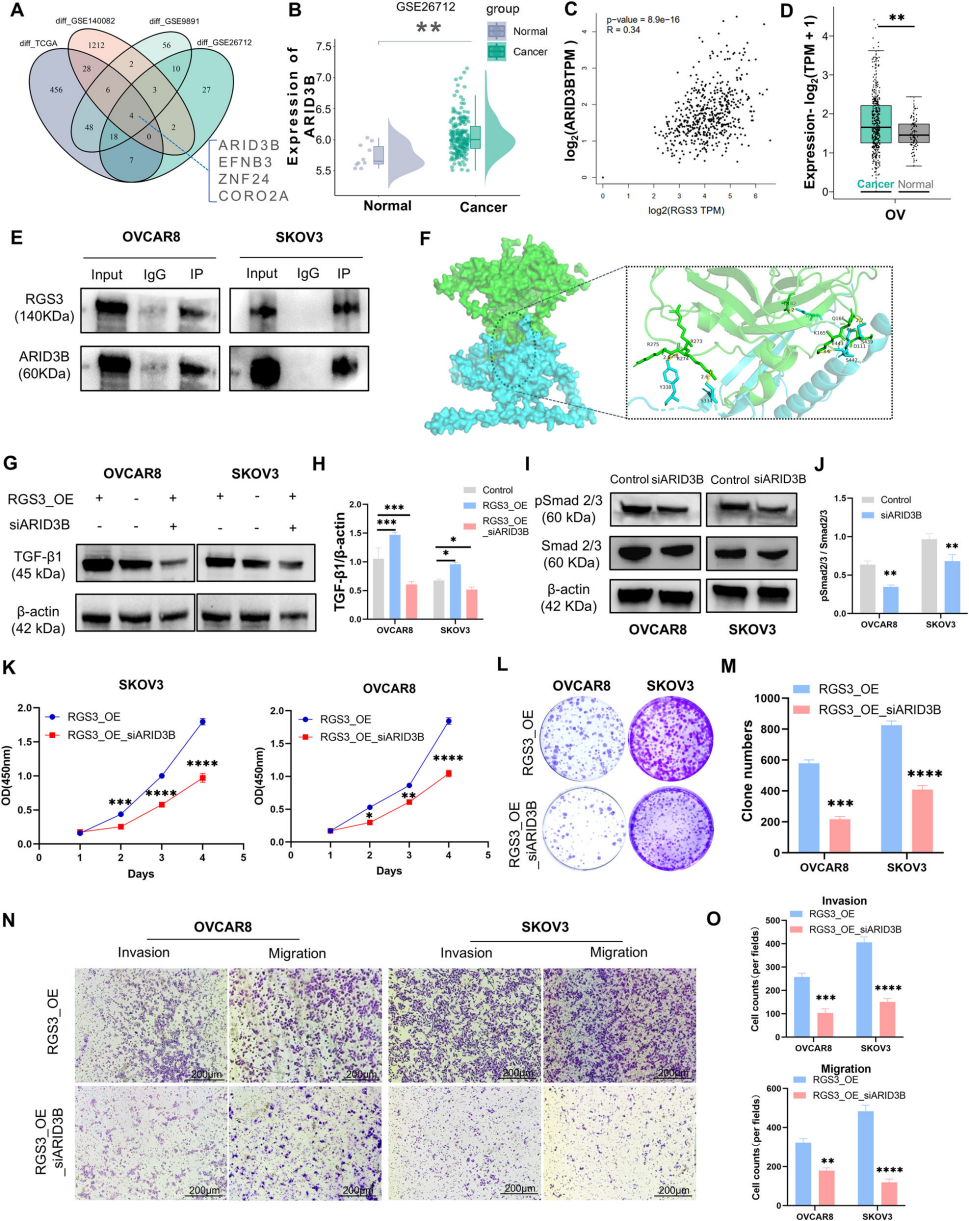
Identifying the downstream effectors of RGS3 in the TGF-β signaling pathway. **A** Identification of common differentially expressed genes across datasets. **B** Differential expression of ARID3B protein in OC tissues compared to normal tissues. **C** Correlation between ARID3B and RGS3 expression via GEPIA-Correlation. **D** GEPIA analysis of ARID3B gene expression in OC vs normal tissues. **E** Co-immunoprecipitation of ARID3B and RGS3 in SKOV3 and OVCAR8 cells. **F** Visualization of RGS3-ARID3B interaction using PyMOL. **G**–**J** Western blot analysis of TGF-β1 and pSmad2/3 expression in RGS3-overexpressing or normal cells with ARID3B knockdown. **K** CCK8 assay measuring cell viability in RGS3-overexpressing or normal cells with ARID3B knockdown. **L**, **M** Colony formation assay images and quantification post-siARID3B transfection. **N**, **O** Transwell assays showing reduced migration and invasion in siARID3B-transfected cells. Data represent mean ± SD of three independent experiments. Differences between the two groups were analyzed using Student’s *t*-test. ^*^P < 0.05, ^**^P < 0.01, ^***^P < 0.001, ^****^P < 0.0001, (scale bar = 200 *μ*m).

To elucidate the role of RGS3 in regulating the TGF-β pathway, we performed bioinformatics analyses on the TCGA-OV, GSE140082, GSE9891, and GSE26712 datasets to identify proteins interacting with RGS3, providing deeper mechanistic insights. Samples were sorted based on RGS3 expression levels, with the median used as a cutoff to simulate RGS3 knockdown and overexpression. We then performed differential expression analysis (adj.Pval < 0.05 and |logFC| > 0.5), considering differentially expressed genes as potential RGS3-interacting proteins. Venn diagram analysis revealed four proteins common across the datasets: ARID3B, EFNB3, ZNF24, and CORO2A (Fig. 7A). Further investigation of the GSE26712 dataset indicated that ARID3B protein levels were significantly higher in OC tissues compared to normal ovarian tissues (Fig. 7B). ARID3B is an evolutionarily conserved transcription factor implicated in normal development, differentiation, cell cycle regulation, and chromatin remodeling [17], as well as in cell immortalization, EMT [18], and tumorigenesis [19].

Correlation analysis conducted via GEPIA demonstrated a positive relationship between ARID3B and RGS3 expression (Fig. 7C), with ARID3B gene expression elevated in OC tissues, consistent with findings from the GSE26712 dataset (Fig. 7D). Confocal microscopy results demonstrated significant colocalization of RGS3 and ARID3B in the cytoplasm of SKOV3 cells (Fig. S2A). Endogenous immunoprecipitation experiments confirmed the direct interaction between RGS3 and ARID3B (Fig. 7E). To investigate the interaction between RGS3 and ARID3B, we conducted protein-protein docking simulations using H-dock software with structures predicted by AlphaFold3. The docking analysis revealed a binding energy of −260.56 kcal/mol, with RGS3 engaging 40 residues and ARID3B interacting with 37 residues (Table S2), visualized within a 5 Å radius using PyMOL (Fig. 7F).

To ascertain the functional significance of ARID3B in mediating RGS3’s effects, we performed rescue experiments by transfecting ARID3B-targeting siRNA into RGS3-overexpressing SKOV3 and OVCAR8 cells to achieve ARID3B knockdown (Fig. S1J, K). As anticipated, ARID3B depletion significantly counteracted the RGS3-induced upregulation of TGF-β1, indicating that ARID3B mediates RGS3’s impact on TGF-β1 expression (Fig. 7G, H). Furthermore, pSmad2/3 levels were found to decrease in siARID3B groups (Fig. 7I, J). Cell viability and proliferation were further assessed using CCK-8 and colony formation assays, revealing that the cell vitality enhancement induced by RGS3 overexpression was counteracted by the downregulation of ARID3B (Fig. 7K, L, M). Additionally, Transwell assays demonstrated that the migration and invasion increase caused by RGS3 overexpression was reversed upon ARID3B knockout (Fig. 7N, O). To further investigate the functional recovery, ARID3B overexpression vectors were transfected into shRGS3 cells. The results indicated that ARID3B overexpression effectively reversed the decline in TGF-β1 and p-Smad2/3 levels induced by RGS3 depletion (Fig. S2B,C). Moreover, cell proliferation, migration, and invasion assays showed that the inhibition of OV cell proliferation, migration, and invasion following RGS3 knockdown was enhanced upon ARID3B upregulation (Fig. S2D-I). In summary, these findings highlight the critical role of ARID3B in mediating the effects of RGS3 on the malignant phenotype of OC cells.

## Discussion

OC is the most lethal gynecological malignancy [20]. Identifying protein biomarkers associated with its proliferation and metastasis is crucial for improving diagnosis and therapeutic targeting [21]. This study explores the biological functions and regulatory mechanisms of potential therapeutic targets in OC.

GPCRs are major drug targets due to their pivotal roles in regulating cellular homeostasis, stress responses, and signal transduction. As key negative regulators of GPCRs, the RGS family offers superior target specificity compared to upstream GPCRs [5]. RGS3, specifically, binds to activated Gαq/11(a member of the Gαq/11 family), inhibiting G11-mediated intracellular calcium mobilization and MAP kinase phosphorylation, thereby deactivating G-protein signaling [10].

Our bioinformatics analysis revealed significant overexpression of RGS3 in OC tissues, further corroborated by IHC showing elevated RGS3 expression in ovarian serous carcinoma and lymphatic metastases. These findings align with reports of RGS3 involvement in other cancers, such as gastric cancer [22], where its overexpression correlates with poor prognosis, and glioma, where RGS3 promotes cell adhesion and metastasis [23]. In our study, both in vitro assays and in vivo models confirmed the pro-proliferative and pro-metastatic effects of RGS3 in OC cells.

Y. Hu et al. developed a five-gene risk model including RGS3 that was significantly enriched in ECM-receptor interaction and TGF-β signaling pathways [9]. Notably, the invasive behavior of epithelial serous OC cells has been closely linked to ECM-receptor interactions [24]. Other studies have indicated that aberrant expression of RGS3 may regulate TGF-β signaling by interfering with Smad protein oligomerization, suggesting a potential regulatory role in OC [25]. Consequently, we investigated the role of RGS3 in the regulation of the TGF-β pathway to clarify the potential mechanisms by which RGS3 regulates OC. TGF-β is a multifunctional cytokine that plays crucial roles in embryonic development, wound healing, tissue homeostasis, and immune regulation [26]. It is also involved in advanced cancer stages by promoting tumor invasion and immune evasion [27]. When TGF-β acts on activated breast epithelial cells expressing Ras, it facilitates EMT and inhibits apoptosis [28]. TGF-β signaling plays an important role in the EMT process in OC [29, 30]. For instance, in gallbladder cancer, TGF-β induces EMT both in vitro [31] and in vivo [32]. Generally, TGF-β1 enhances ECM production by stimulating collagen and fibronectin synthesis and is involved in EMT [33]. TGF-β is typically considered a promoter of tumor invasion and metastasis, particularly in advanced tumors. Elevated levels of TGF-β and its receptors are associated with disease progression and poor prognosis in cancers such as breast [34], pancreatic [35], and gastric cancer [36]. Smad2/3 signaling has been identified as a critical promoter of OC initiation and metastasis, further implicating the role of TGF-β signaling in OC progression [37].

EMT-associated proteins like N-cadherin, vimentin, and Snail are critical markers of cancer metastasis [38]. EMT has also been associated with drug resistance, as increased EMT markers correlate with resistance to paclitaxel in OC epithelial cell lines [39]. Furthermore, cancer-related inflammation can induce EMT, linking inflammation to cancer progression and fibrosis [40]. Our data demonstrated that knockdown of RGS3 significantly decreased TGF-β1 expression and EMT markers, while overexpression of RGS3 increased these proteins, implicating the RGS3/TGF-β/EMT axis in OC progression. TGF-β1 encodes a secreted ligand of the TGF-β superfamily, which is involved in cancer development and progression [41]. In OC, TGF-β1 regulates cell proliferation, EMT, stem cell phenotype, and metastasis. Blocking the TGF-β1 pathway can inhibit self-renewal, migration, and invasion of OC stem cells [42]. Ramos et al. reported that TGF-β1 regulates cell invasion by upregulating CAF-derived versican in OC [43].

ARID3B, a member of the AT-rich interactive Domain protein family, plays crucial roles in embryonic development and tumor growth [44]. Abnormal expression of ARID proteins is associated with tumorigenesis [45]. ARID3B is overexpressed in neuroblastoma and serous OC, and its overexpression is linked to disease recurrence [46–48]. Research has shown that ARID3B induces Wnt receptor FZD5, which is important for enhancing tumor cell adhesion and may contribute to metastasis [49]. In this study, ARID3B was identified as a direct interacting protein of RGS3, substantiated through computational modeling and co-IP experiments. Notably, the silencing of ARID3B effectively reversed the upregulation of TGF-β1 and p-Smad2/3 expression induced by RGS3 overexpression. Further proliferation, migration, and invasion assays also confirmed that silencing ARID3B effectively reversed the promotion of tumor cell proliferation and metastatic potential by RGS3 overexpression. This suggests that RGS3 may regulate the expression of the TGF-β pathway through its interaction with ARID3B, contributing to the proliferation and metastasis of OC. Additional rescue experiments demonstrated that ARID3B overexpression could reverse the inhibition of OC cell proliferation and metastasis induced by RGS3 silencing. Our findings demonstrate that RGS3 promotes the proliferation and metastasis of OC cells by targeting the TGF-β1/p-Smad2/3 axis and inducing EMT transformation. One limitation of our findings was that the interaction between RGS3 and ARID3B lacks validation in clinical samples. The inclusion of data from patient samples, such as immunohistochemical analysis or correlation studies of RGS3 and ARID3B expression, would enhance the translational relevance of the findings.

## Conclusion

In summary, our study suggested that RGS3 is upregulated in OC tissues and cell lines. RGS3 depletion effectively inhibits OC cell proliferation and metastasis. RGS3 exerts its effects through the TGF-β signaling pathway and its mediation of EMT through its direct interaction with ARID3B. Taken together, our results indicate an inhibitory effect of RGS3 knockdown on tumor development and suggest that targeting RGS3 could be a novel therapeutic strategy for treating OC.

## Methods

### Immunohistochemistry (IHC) staining assay and clinical tissue specimens

A total of 31 ovarian tissue samples were collected from the Department of Obstetrics and Gynecology at the First Affiliated Hospital of Soochow University. The control group consisted of samples confirmed to be histologically normal. IHC assays were performed according to a previously established study [50]. Staining results were evaluated based on both intensity and the percentage of positively stained cells. The study was approved by the Ethics Committee of the First Affiliated Hospital of Soochow University (approval number: 2024-483) in compliance with the Declaration of Helsinki. All participants provided written informed consent.

### OC cell culture

Human OC cell lines (SKOV3, HO8910, OVCAR8, A2780) and the normal ovarian epithelial cell line (IOSE80) were obtained from the Cell Bank of Type Culture Collection of the Chinese Academy of Sciences. Cells were cultured in RPMI 1640 medium (HyColony, Logan, UT, USA) with 10% fetal bovine serum (Procell Life Science & Technology Co., Ltd., China) and 1% penicillin-streptomycin (Beyotime, China) and maintained in a humidified incubator at 37 °C with 5% CO_2_.

### Bioinformatics analysis

Gene expression profiling interactive analysis (GEPIA) (http://gepia.cancer-pku.cn/) [51], which integrates data from The Cancer Genome Atlas (TCGA) and the genotype-tissue expression (GTEx) projects, includes RNA sequencing expression data from 9,736 tumor and 8,587 normal samples, was employed to evaluate the transcriptional levels of RGS3 and ARID3B across cancers, with a particular focus on OC. Furthermore, ARID3B mRNA expression was analyzed in the GSE26712 dataset from the Gene Expression Omnibus (GEO), containing 185 primary ovarian tumor samples and 10 normal ovarian surface epithelium samples. TCGA RGS3-related co-expressed genes were identified from TCGA, following stringent criteria (|Spearman’s correlation coefficient | > 0.3, *P* < 0.001) [52] and analyzed via Gene Ontology (GO) and Kyoto Encyclopedia of Genes and Genomes (KEGG).

In GSE26712, samples were ranked by RGS3 expression, with high and low groups defined by the median, followed by Gene Set Enrichment Analysis (GSEA). Interaction proteins of RGS3 were explored using datasets from TCGA, GSE26712, GSE9891 (comprising 285 OC samples), and GSE140082 (comprising 380 OC samples), with differential gene expression analysis (adj.Pval < 0.05, |logFC | > 1) after the same high- and low-expression grouping method mentioned above. Interaction proteins were hypothesized from the overlap of differentially expressed genes.

### Simulation analysis of Interacting Proteins

Protein sequences for RGS3 and ARID3B were retrieved from the UniProt database (https://www.uniprot.org/) [53]. Structural models of these proteins were generated using AlphaFold3 (https://alphafoldserver.com/) [54]. Protein-protein docking was then conducted using the H-dock server (http://hdock.phys.hust.edu.cn/) [55], generating 100 possible binding conformations. The results were ranked by binding energy, and the lowest-energy conformation was selected for detailed analysis. PyMOL was used for the visualization of docking results [56].

### Transient transfection

SKOV3 and OVCAR8 cells were transfected with siRNA targeting RGS3 (siRGS3), ARID3B(siARID3B), and a non-targeting control (siNC), all obtained from GenePharma (Shanghai, China). The sequences used were:

siRGS3:

Sense: 5ʹ-CCAAGGACAMGAAGAACAATT-3ʹ

Antisense: 5ʹ-UUGUUCUUCAUGUCCUUGGTT-3ʹ

siARID3B:

Sense: 5ʹ-GGAGCAUUAACAUGUCUGUTT-3ʹ

Antisense: 5ʹ-ACAGACAUGUUAAUGCUCCTT-3ʹ

Normal control (non-target siRNA):

Sense: 5ʹ-UUCUCCGAACGUGUCACGUTT-3ʹ

Antisense: 5ʹ-ACGUGACACGUUCGGAGAATT-3ʹ

For overexpression studies, SKOV3 and OVCAR8 cells were transfected with the pcDNA3.1-RGS3-Homo plasmid, also purchased from GenePharma. A control vector was used as a comparison. Transfections were carried out using Lipofectamine 3000 (Invitrogen, Carlsbad, CA, USA) following the manufacturer’s instructions.

### Lentiviral infection

Lentiviral vectors containing RGS3 short hairpin RNA (shRNA) and RGS3 overexpression constructs were synthesized by General Biosystems (Anhui, China). Control cells were infected with an empty vector. SKOV3 and OVCAR8 cells were transduced with lentiviral particles at a multiplicity of infection (MOI) of 10. After 72 h, cells underwent selection with puromycin (2 μg/mL) for 7–10 days. Transduction efficiency was monitored by GFP fluorescence and confirmed via Western blot.

### RNA isolation and real-time quantitative PCR (RT-qPCR) analysis

Total RNA was extracted using TRIzol reagent (Invitrogen). cDNA synthesis was performed using 1 μg of RNA per sample with the HiScript III RT SuperMix Kit (#R323, Vazyme, Nanjing, China). The PCR primers were:

RGS3:

Forward: 5ʹ-GCAAGCTCAATTATAAGCCTCC-3ʹ

Reverse: 5ʹ-CTTCCAGATCTCCAGTAAGGTC-3ʹ

GAPDH:

Forward: 5ʹ-CACCCACTCCTCCACCTTTGAC-3ʹ

Reverse: 5ʹ-GTCCACCACCCTGTTGCTGTAG-3ʹ

RT-qPCR was conducted using ChamQ Universal SYBR qPCR Master Mix (Vazyme) on a CFX96 Touch Real-Time PCR System (Bio-Rad, CA, USA). Cycling conditions included initial denaturation at 95 °C for 5 min, followed by 40 cycles of 95 °C for 10 s and 60 °C for 30 s. Data were analyzed using the 2^−ΔΔCt^ method with GAPDH as the endogenous control.

### Cell counting kit-8 (CCK-8) assay

Transiently transfected SKOV3 and OVCAR8 cells were seeded at a density of 2000 cells per well and incubated at 37 °C with 5% CO_2_. After incubation, 10 µL of CCK-8 solution (GlpBio Technology Inc., USA) was added to each well. The cells were further incubated for 1 h, and absorbance at 450 nm was measured using a Multiskan FC microplate reader (Thermo Scientific, USA) at 24,48,72, and 96 h post-transfection. Cell viability was determined based on the absorbance values.

### Transwell migration and invasion assay

Migration and invasion assays were performed using 24-well Transwell chambers with 8 µm pore inserts, either coated with Matrigel (for invasion) or uncoated (for migration) (BD Biosciences). Cells were suspended in serum-free medium at 8 × 10^4^ cells/mL, and 300 µL of the suspension was added to the upper chamber, while 500 µL of medium containing 20% FBS was placed in the lower chamber. The cells were incubated at 37 °C in 5% CO_2_ for 24 h (migration) or 36 h (invasion). Following incubation, non-migrating cells were removed from the upper surface of the inserts, and invasive cells on the lower surface were fixed with 4% paraformaldehyde (Sinopharm Chemical Reagent Co., Ltd., China) and stained with 0.5% crystal violet (Beyotime Biotechnology, Shanghai, China). The stained cells were then visualized under a microscope (Leica Microsystems, Germany).

### Apoptosis assay

SKOV3 and OVCAR8 cells were seeded in 6-well plates and transfected with siRGS3 or the corresponding plasmid. The following day, cells were stained with PI/RNase buffer (BD Biosciences PharMingen, USA). Apoptosis was assessed using the Annexin V FITC Apoptosis Detection Kit (BD Biosciences PharMingen, No. 556547) with flow cytometry to distinguish live, early, and late apoptotic cells.

### Western blot analysis

Cells were lysed in 1% SDS buffer (#P0013G, Beyotime) and sonicated on ice for 30 s. Protein concentration was determined using a BCA protein assay kit (#P0009, Beyotime). Proteins were separated via 10% SDS-polyacrylamide gel electrophoresis (SDS-PAGE) gels (#PG110, #PG112, #PG113, Epizyme Biotech), transferred to polyvinylidene fluoride (PVDF) membranes (#IPVH00010, Merck Millipore, Germany), and blocked with 5% non-fat milk. Membranes were incubated overnight with primary antibodies (RGS3, ARID3B, TGF-β1, p-Smad2/3, Smad2/3, E-cadherin, Snail, N-cadherin, and Vimentin), followed by HRP-conjugated secondary antibodies. Protein bands were visualized with a ChemiDoc™ MP system (Bio-Rad). Uncropped Western blot images are shown in the Supplementary File.

### Colony formation assay

Transfected cells (1000 cells/well) were seeded in 6-well plates and incubated for 10–14 days. Colonies were fixed with 4% paraformaldehyde, stained with 0.5% crystal violet (Beyotime Institute of Biotechnology), and observed under a microscope (Leica Microsystems, Germany) at 100× magnification. Colonies with more than 50 cells were counted, and data were analyzed using ImageJ.

### Co-immunoprecipitation (Co-IP)

To examine protein-protein interactions, SKOV3 and OVCAR8 cells transfected with RGS3-Flag and ARID3B-myc plasmids were lysed using IP lysis buffer (#IF0958, Engibody). Five micrograms of each antibody were conjugated to magnetic beads and incubated overnight at 4 °C with rotation. Equal amounts of cell lysates were then incubated with the antibody-conjugated beads. The beads were collected using a magnetic separator and washed three times. The proteins bound to the beads were eluted with 6 μL Sample Loading Buffer and 24 μL Elution Buffer. The eluted proteins were analyzed by Western blotting.

### Immunofluorescence (IF) analysis

SKOV3 cells were treated with designated primary antibodies at 4 °C overnight, followed by incubation with the appropriate secondary antibodies at 37 °C for 2 h in a dark environment. Subsequently, nuclei were visualized by staining cells with DAPI for 10 min. Fluorescence imaging was conducted by confocal microscopy (Nikon A1R, Japan) in the dark setting.

### In vivo assay

Female Non-Obese Diabetic Severe Combined Immunodeficient (NOD-SCID) mice (3–5-weeks-old) were obtained from GemPharmatech Co., Ltd (Jiangsu, China). All experimental protocols were approved by the Ethics Committee of Soochow University (approval no. 202405A0007) and were conducted in compliance with institutional animal use regulations. Mice were housed in specific pathogen-free (SPF) conditions, with 35–40% relative humidity, a 12-h light/dark cycle, and ad libitum access to standard food and water. At the experimental endpoint, mice were euthanized by cervical dislocation. To evaluate tumor growth and metastasis, 5 × 10^5^ cells (OVCAR8-shNC, OVCAR8-shRGS3, OVCAR8-Control, and OVCAR8-RGS3-overexpressing) were injected either subcutaneously or intraperitoneally (i.p.). Mice receiving subcutaneous injections were euthanized 4 weeks post-injection, after which tumors were excised and weighed. Tumor dimensions were measured every 6 days using a Vernier caliper, and tumor volume was calculated using the formula length/2 × width^2^, with average length and width values. In the i.p. injection model, visceral organs (including the liver, intestines, mesentery, kidneys, ovaries, and diaphragm) were examined for metastases 7 weeks post-injection, and metastasized tissues were collected. IHC analysis was performed on both primary tumors and metastases. To quantify the intensity of positive signal expression, images were captured using a microscope (Leica Microsystems, Germany) and converted into TIFF (Tagged Image File Format) format. Three randomly selected fields (40× objective magnification) per tissue section were scanned and analyzed using ImageJ, version 1.54 (National Institutes of Health, Bethesda, MD). The integrated optical density (IOD) of each image was measured after correcting for optical density, with segmentation set at a constant level to enable detection of positive immunostaining. Data are presented as mean ± SD of IOD readings obtained from three fields. All animal procedures adhered to the “Laboratory Animal—Guidelines for Ethical Review of Animal Welfare” (GB/T 35892–2018).

### Statistical analysis

Data were analyzed using Student’s *t*-test or one-way/two-way analysis of variance (ANOVA) as appropriate. Pearson’s correlation coefficient was used for correlation analyses. Statistical significance for Venn diagrams was assessed using the chi-square test. All experiments were performed in triplicate unless otherwise specified. Data analysis and visualization were conducted using GraphPad Prism 9 (GraphPad Software, San Diego, CA), and results are expressed as mean ± SD. Statistical significance is denoted in the figures with asterisks: ^*^P < 0.05, ^**^P < 0.01, ^***^P < 0.001, ^****^P < 0.0001.

## Supplementary information

Figure S1

Figure S2

Table S1

Table S2

Supplementary Information

Uncropped Western Blots

## Data Availability

The data that supports the findings of this study are available in the article and the supplementary materials
