# Supplementary figures and images for: RGS3 acts as a tumor promoter by facilitating the regulation of the TGF-β signaling pathway and promoting EMT in ovarian cancer
Source: Cell Death Discov. 2025 Jun 2;11:262. doi: 10.1038/s41420-025-02536-3 (PMC12130528; doi:10.1038/s41420-025-02536-3)

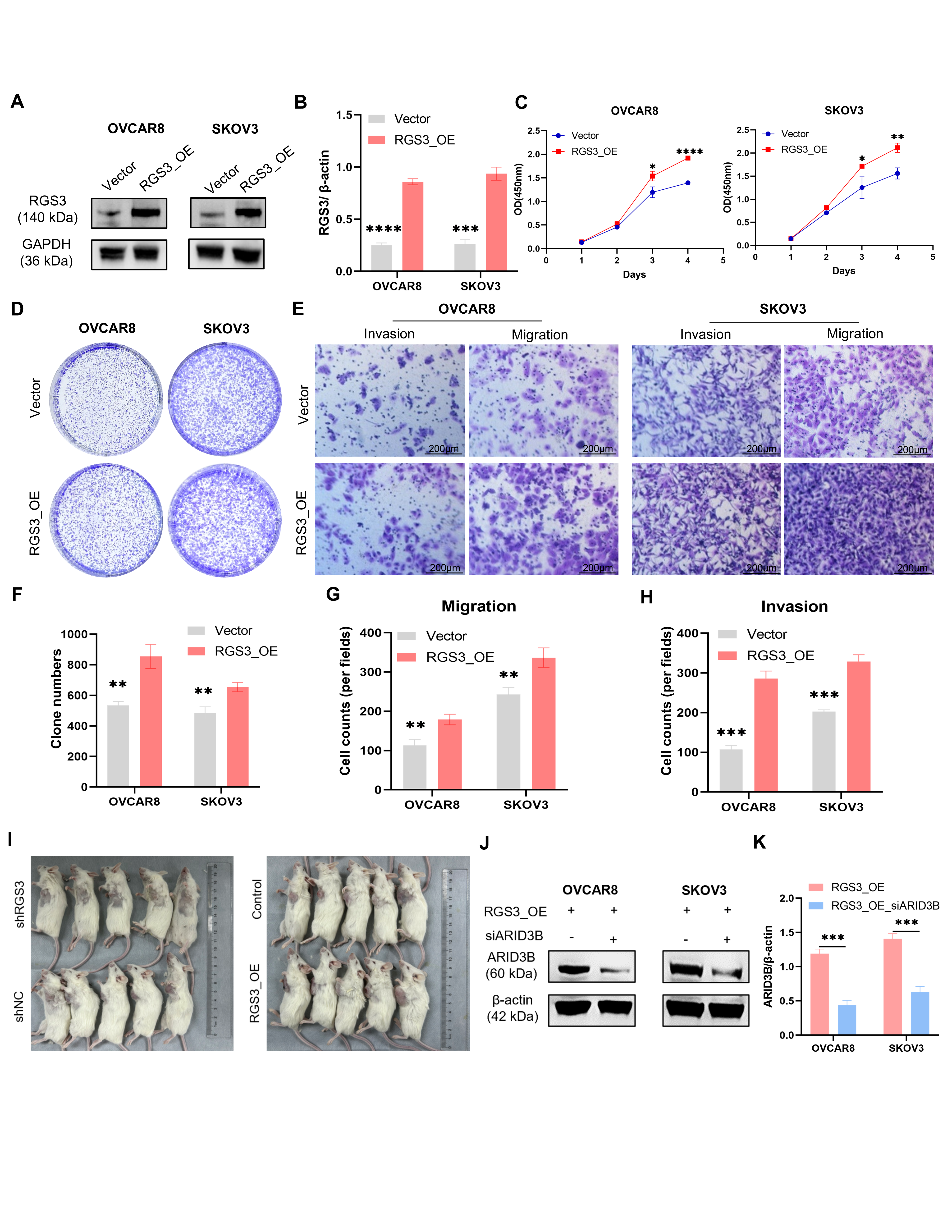

Supplement: Supplementary file 1 — Figure S1 [file 41420_2025_2536_MOESM1_ESM.tif]

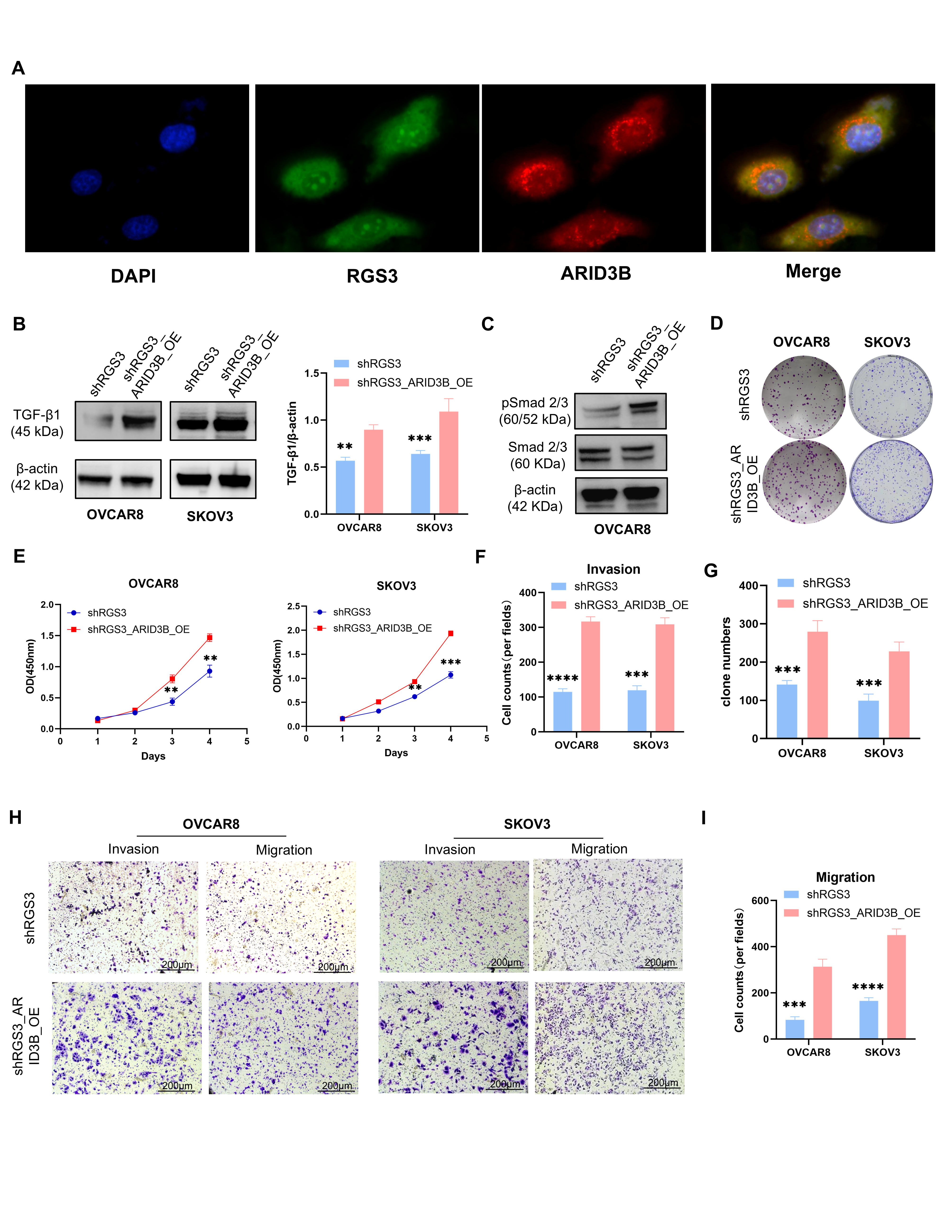

Supplement: Supplementary file 2 — Figure S2 [file 41420_2025_2536_MOESM2_ESM.tif]

Fig 1 .

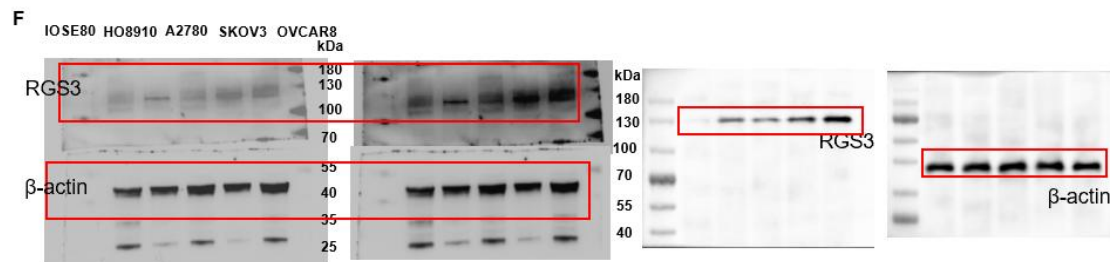

Fig 2, S1.

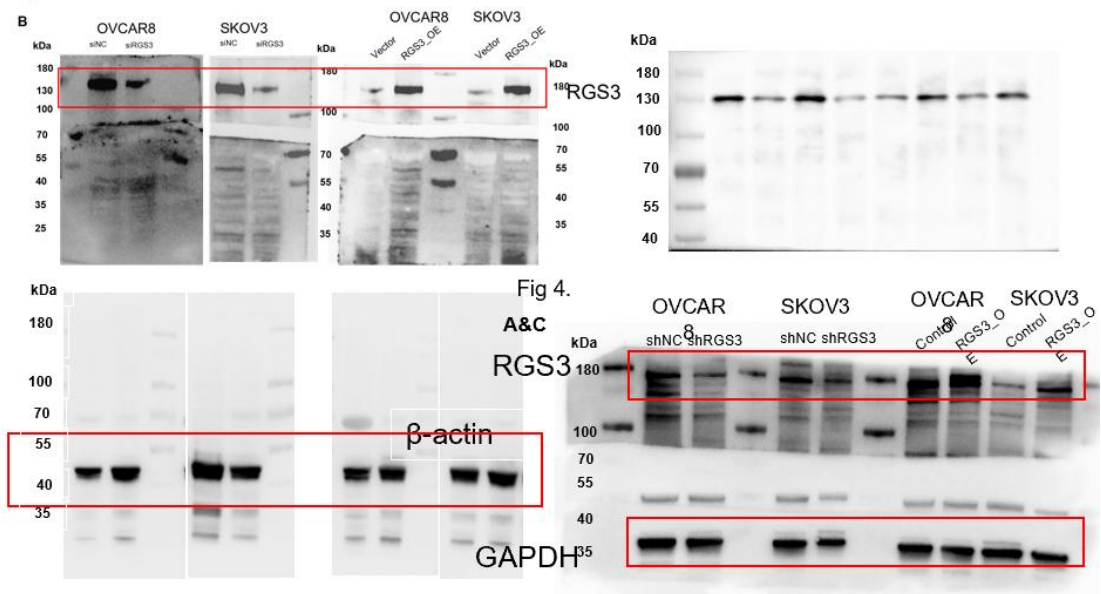

Fig 6.

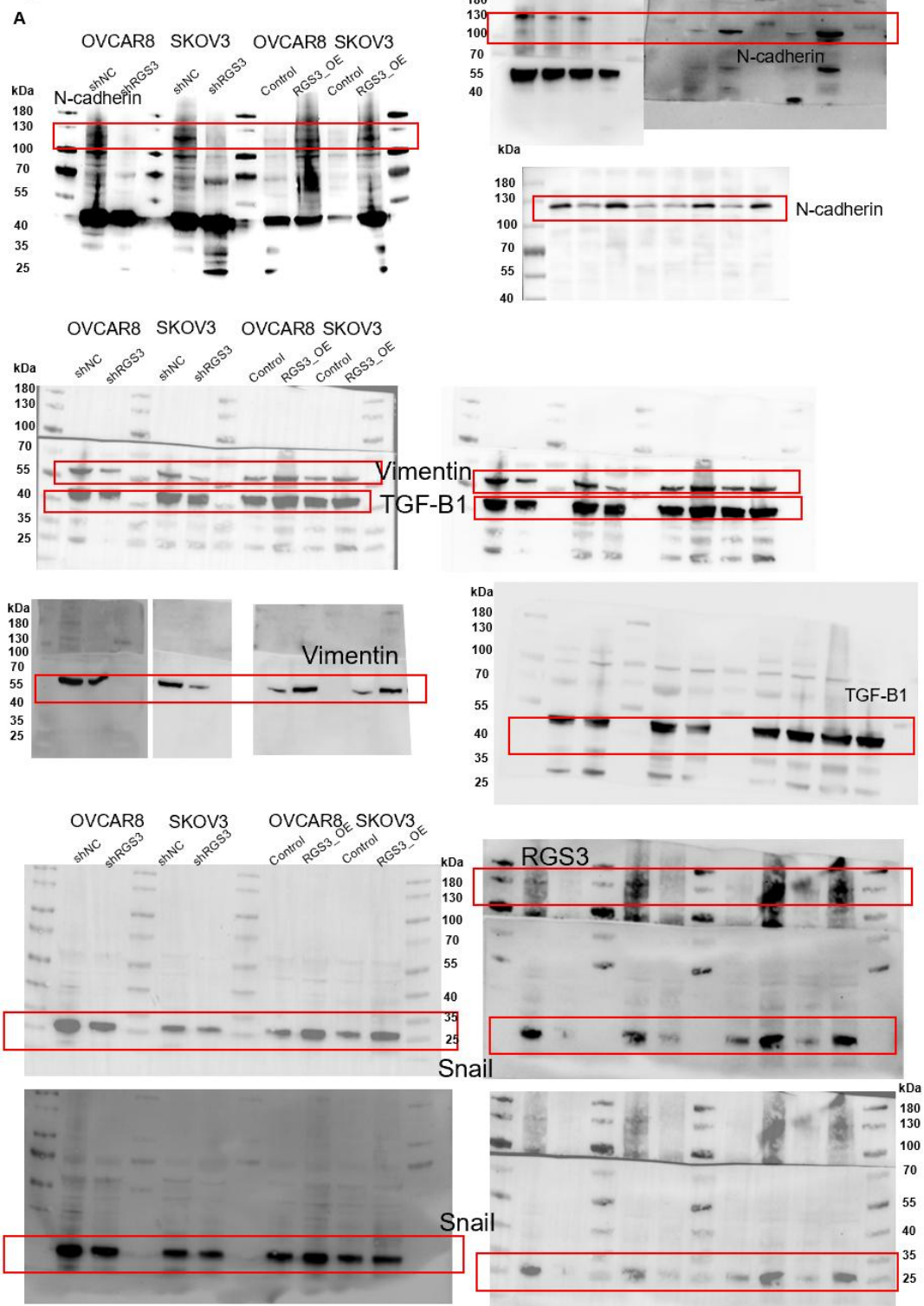

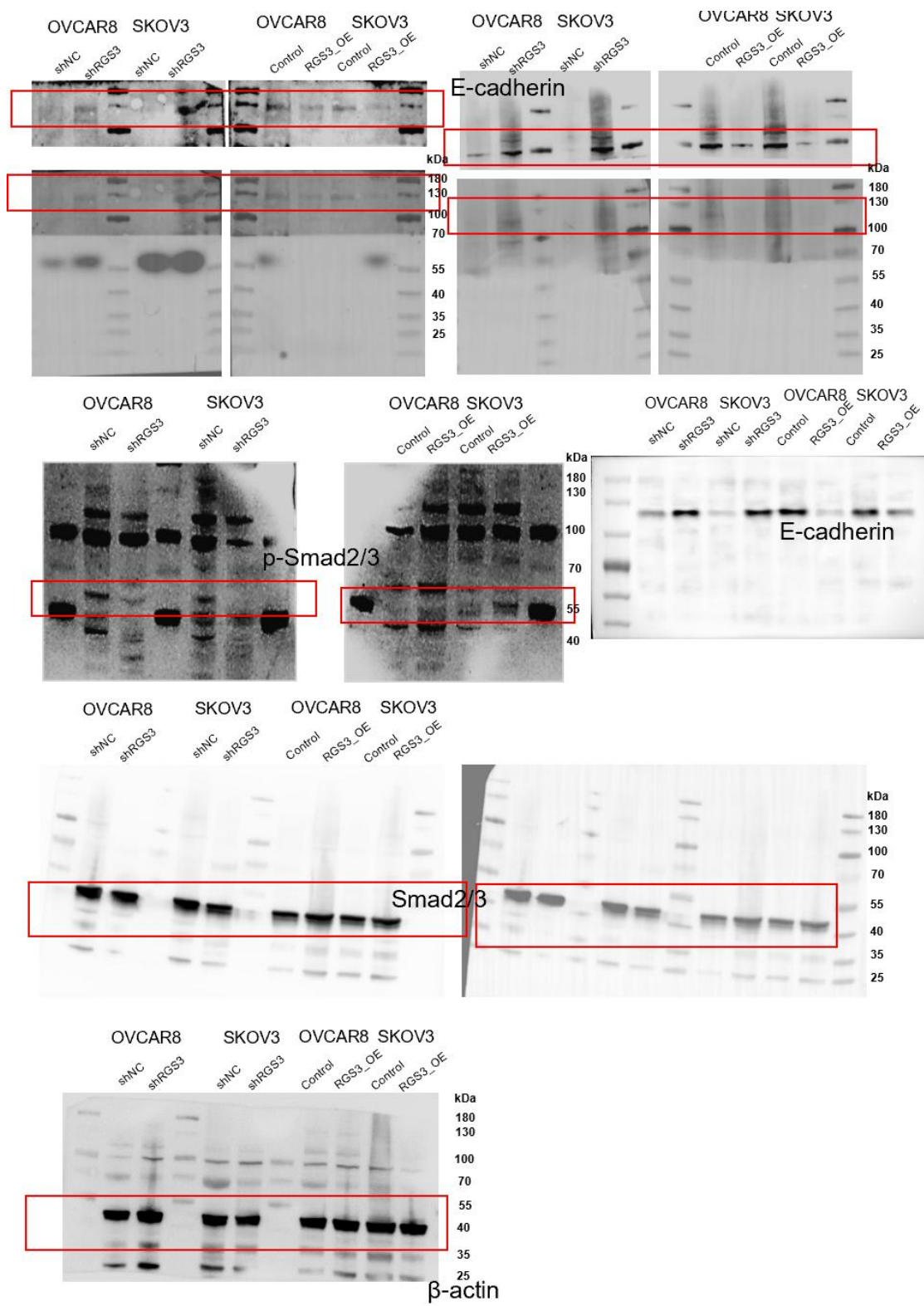

Fig 7E.

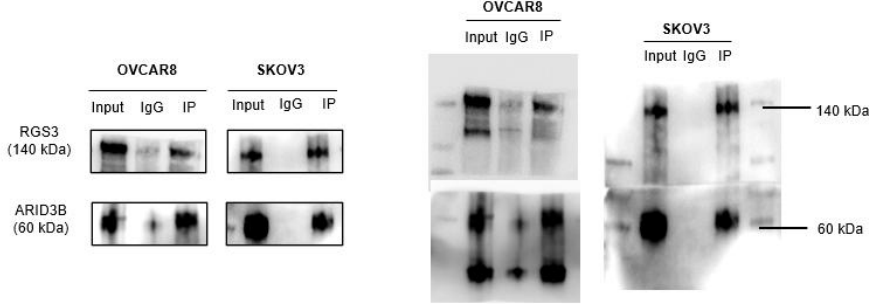

Fig 7.G

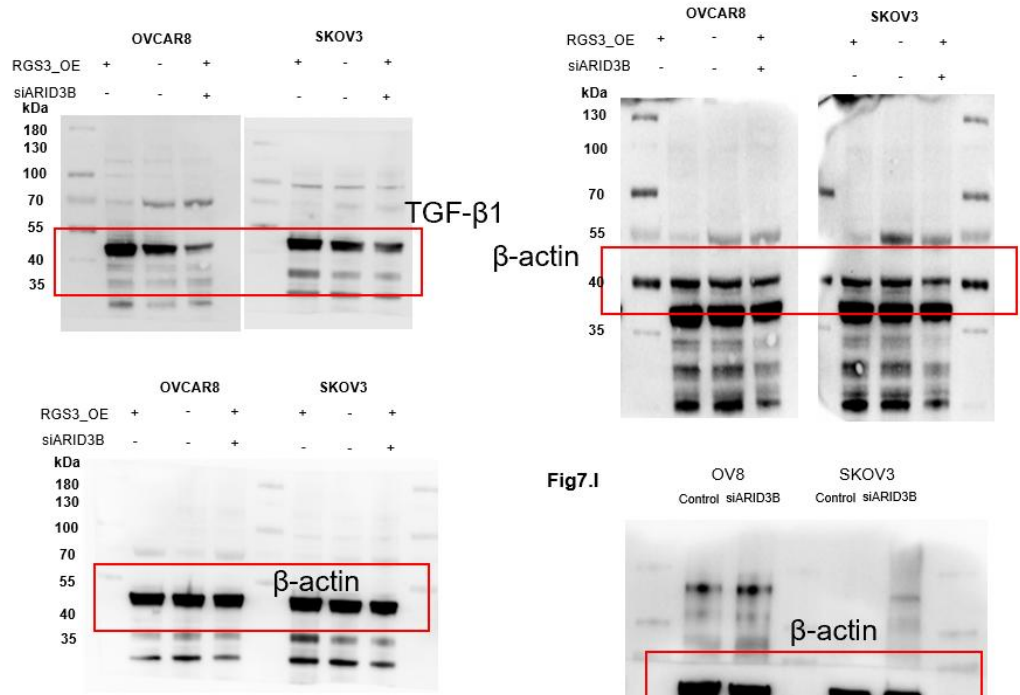

Fig7.I

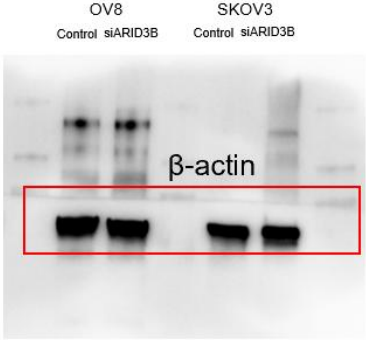

Fig7.I

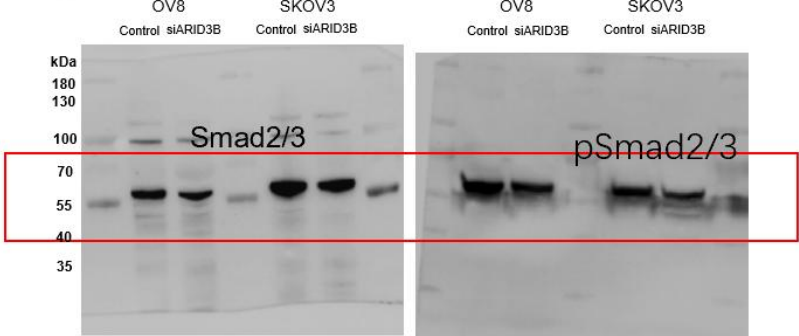

Fig.S2B

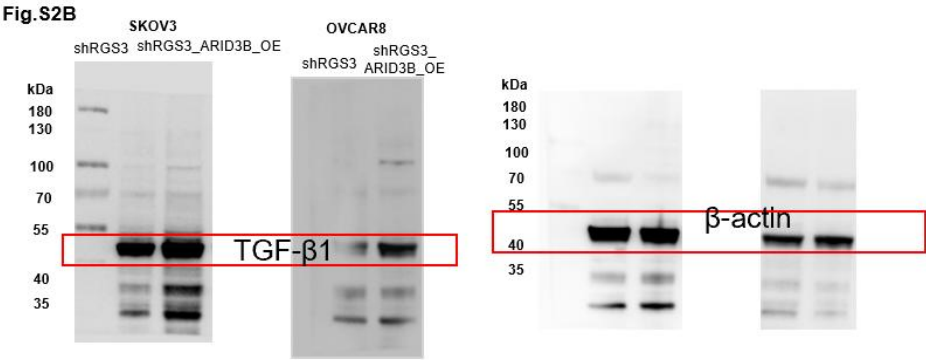

Fig S1.J

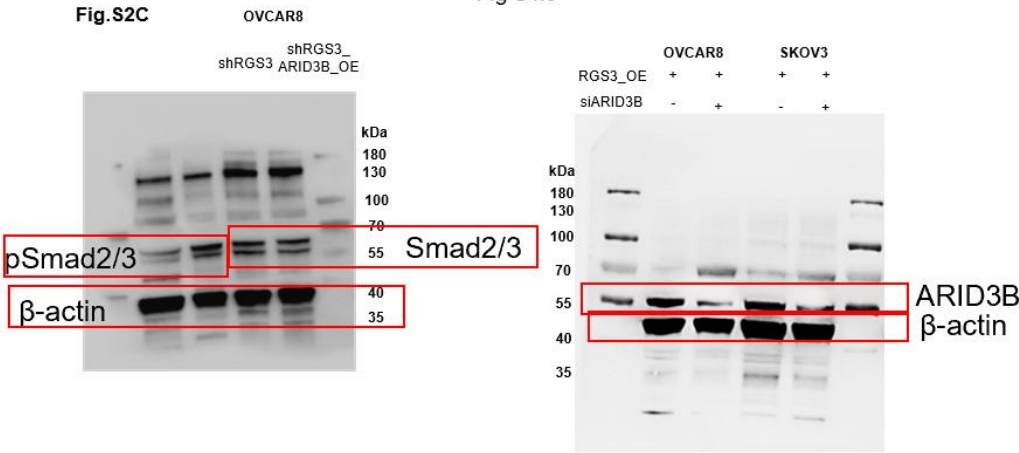

Supplement: Supplementary file 6 — Uncropped Western Blots [file 41420_2025_2536_MOESM6_ESM.pdf]
