# Supplementary material for: RGS3 acts as a tumor promoter by facilitating the regulation of the TGF-β signaling pathway and promoting EMT in ovarian cancer
Source: Cell Death Discov. 2025 Jun 2;11:262. doi: 10.1038/s41420-025-02536-3 (PMC12130528; doi:10.1038/s41420-025-02536-3)
Supplement: Supplementary file 3 — Table S1 [file 41420_2025_2536_MOESM3_ESM.docx]

**Table S1.** Relationship between RGS3 expression level and clinical characteristic parameters of patients

| Clinical parameters | Cases (n) | RGS3 expression (%) | | p-value |
| --- | --- | --- | --- | --- |
|  |  | Low | High |  |
| Age (years) |  |  |  |  |
| ≥50 | 27 | 10 | 17 | 0.553 |
| <50 | 4 | 1 | 3 |  |
| FIGO staging |  |  |  |  |
| Ⅰ | 1 | 1 | 0 | 0.3703 |
| Ⅱ | 2 | 1 | 1 |  |
| Ⅲ | 4 | 2 | 2 |  |
| Ⅳ | 6 | 1 | 5 |  |
| T stage |  |  |  |  |
| T1 | 1 | 1 | 0 | 0.2956 |
| T2 | 4 | 2 | 2 |  |
| T3 | 8 | 2 | 6 |  |
| N stage |  |  |  |  |
| N1 | 9 | 4 | 5 | >0.99 |
| N0 | 4 | 1 | 3 |  |
| M stage |  |  |  |  |
| M1 | 8 | 4 | 4 | 0.5649 |
| M0 | 5 | 1 | 4 |  |
| Pathology diagnosis |  |  |  |  |
| Normal ovarian tissue | 5 | 5 | 0 | 0.0002 |
| Ovarian serous carcinoma | 13 | 6 | 7 |  |
| Metastasis serous carcinoma from ovary | 13 | 0 | 13 |  |
