# Supplementary material for: RGS3 acts as a tumor promoter by facilitating the regulation of the TGF-β signaling pathway and promoting EMT in ovarian cancer
Source: Cell Death Discov. 2025 Jun 2;11:262. doi: 10.1038/s41420-025-02536-3 (PMC12130528; doi:10.1038/s41420-025-02536-3)
Supplement: Supplementary file 4 — Table S2 [file 41420_2025_2536_MOESM4_ESM.docx]

**Table S2.** Pairing residues of RGS3-ARID3B interaction interface

| Receptor-ligand interface residue pair(s) | residue spacing（Å） |
| --- | --- |
| 105A-518A | 3.968 |
| 109A-443A | 2.805 |
| 110A-443A | 4.208 |
| 110A-447A | 4.5 |
| 111A-440A | 4.455 |
| 111A-443A | 3.323 |
| 111A-444A | 4.627 |
| 111A-447A | 3.063 |
| 112A-443A | 2.798 |
| 112A-446A | 3.518 |
| 112A-447A | 4.08 |
| 112A-450A | 3.504 |
| 113A-447A | 2.989 |
| 113A-450A | 2.378 |
| 114A-450A | 4.152 |
| 114A-454A | 4.835 |
| 115A-450A | 3.001 |
| 115A-454A | 3.436 |
| 116A-454A | 3.278 |
| 117A-511A | 4.396 |
| 117A-513A | 3.094 |
| 118A-510A | 3.854 |
| 119A-453A | 4.918 |
| 119A-454A | 3.065 |
| 119A-457A | 3.324 |
| 119A-458A | 3.991 |
| 121A-501A | 4.887 |
| 122A-457A | 2.792 |
| 122A-466A | 2.898 |
| 122A-497A | 4.851 |
| 122A-499A | 2.819 |
| 122A-501A | 3.415 |
| 123A-457A | 3.529 |
| 123A-460A | 3.991 |
| 123A-464A | 3.055 |
| 123A-466A | 4.842 |
| 123A-501A | 4.629 |
| 125A-460A | 4.774 |
| 129A-123A | 3.365 |
| 131A-120A | 3.418 |
| 131A-123A | 3.137 |
| 132A-335A | 4.852 |
| 132A-336A | 4.551 |
| 133A-116A | 3.365 |
| 133A-119A | 4.782 |
| 133A-120A | 4.598 |
| 134A-120A | 2.212 |
| 136A-116A | 4.399 |
| 136A-335A | 4.544 |
| 165A-438A | 4.096 |
| 165A-442A | 3.38 |
| 165A-446A | 3.639 |
| 166A-435A | 3.599 |
| 166A-438A | 4.764 |
| 166A-439A | 2.45 |
| 167A-434A | 4.178 |
| 167A-435A | 1.543 |
| 167A-438A | 4.261 |
| 168A-435A | 2.806 |
| 169A-435A | 4.348 |
| 182A-513A | 3.221 |
| 184A-518A | 2.787 |
| 226A-435A | 4.583 |
| 228A-433A | 4.877 |
| 228A-435A | 4.522 |
| 229A-436A | 3.953 |
| 231A-436A | 4.324 |
| 231A-439A | 3.863 |
| 234A-446A | 4.299 |
| 257A-335A | 4.919 |
| 261A-446A | 2.705 |
| 262A-446A | 3.655 |
| 262A-449A | 2.76 |
| 262A-450A | 3.346 |
| 262A-453A | 4.948 |
| 273A-338A | 2.775 |
| 274A-331A | 4.493 |
| 274A-334A | 2.585 |
| 274A-335A | 2.957 |
| 274A-338A | 3.493 |
| 275A-338A | 2.631 |
