# Supplementary material for: RGS3 acts as a tumor promoter by facilitating the regulation of the TGF-β signaling pathway and promoting EMT in ovarian cancer
Source: Cell Death Discov. 2025 Jun 2;11:262. doi: 10.1038/s41420-025-02536-3 (PMC12130528; doi:10.1038/s41420-025-02536-3)
Supplement: Supplementary file 5 — Supplementary Information [file 41420_2025_2536_MOESM5_ESM.docx]

Supplementary Information for

**RGS3 acts as a tumor promoter by facilitating the regulation of the TGF-β signaling pathway and promoting EMT in ovarian cancer**

Zizhao Wang^1,2,3#^, Huating Sun^1,2,3#^, Shunpeng Zhu^1,2^, Fang Wang^1^, Quan Li^1,2^, Jinhua Zhou^1^*

**Figure. S1 RGS3 overexpressing promotes ovarian cancer proliferation and metastasis.** (A, B) Western blot confirmation of RGS3_OE transfection efficiency. (C) CCK8 assay measuring cell viability in SKOV3 and OVCAR8 cells post-RGS3_OE transfection. (D, F) Colony formation assay images and quantification post-RGS3_OE transfection. (E, G, H) Transwell assays showing reduced migration and invasion in RGS3_OE-transfected cells. (I) Xenograft tumor growth and size comparison between RGS3 knockdown and overexpression groups. (J, K) Western blot confirmation of siARID3B transfection efficiency in SKOV3 and OVCAR8 cells. Data represent mean ± SD of three independent experiments. Differences between two groups were analyzed using Student’s t-test. **P < 0.05, **P < 0.01, ***P < 0.001,* (Scale bar = 200 μm).

**Figure. S2 Identifying the downstream effectors of RGS3 in TGF-β signaling pathway.** (A) Double-label immunofluorescence staining for the intracellular localization of RGS3 and ARID3B in SKOV3 cells. (B, C) Western blot analysis of TGF-β1 and pSmad2/3 expression in ARID3B-overexpressing or normal cells with RGS3 knockdown. (E) CCK8 assay measuring cell viability in ARID3B-overexpressing or normal cells with RGS3 knockdown. (D, G) Colony formation assay images and quantification post-ARID3B_OE transfection. (F, H, I) Transwell assays showing higher migration and invasion in ARID3B_OE-transfected cells. Data represent mean ± SD of three independent experiments. Differences between two groups were analyzed using Student’s t-test. **P < 0.05, **P < 0.01, ***P < 0.001, ****P < 0.0001* (Scale bar = 200 μm).

**Table S1.** Relationship between RGS3 expression level and clinical characteristic parameters of patients

**Table S2.** Pairing residues of RGS3-ARID3B interaction interface

**Docking Score:** -260.56 kcal/mol

**Confidence Score:** 0.9013

**Ligand rmsd (Å):** 80.94

**Docking Score**: The docking scores are calculated by ITScorePP or ITScorePR. A more negative docking score means a more possible binding model.

**Confidence Score**: to indicate the binding likeliness of two molecules as follows. Roughly, when the confidence score is above 0.7, the two molecules would be very likely to bind.

- Confidence_score = 1.0/[1.0+e^0.02*(Docking_Score+150)^]

**Ligand rmsd**: The ligand RMSDs are calculated by comparing the ligands in the docking models with the input or modeled structures.

**Interface redidues**: The interface information for each model includes all the residue pairs within 5.0 Å between the receptor and the ligand for the corresponding model. Users can click to check/dowload the files for different models.

**RGS3 short hairpin RNA (shRNA) Sequence**

ATGGAGTGGCTAAGCCCTGATATCGCTCTGCCCAGAAGAGATGAGTGGACTCAAACTTCTCCAGCCAGGAAGAGGATCACGCATGCCAAAGTCCAGGGTGCAGGTCAGCTGAGGCTGTCCATTGATGCCCAGGACCGGGTTCTGCTGCTTCACATTATAGAAGGTAAAGGCCTGATCAGCAAACAGCCTGGCACCTGTGATCCGTATGTGAAGATTTCTTTGATCCCTGAAGATAGTAGACTACGCCACCAGAAGACGCAGACCGTTCCAGACTGCAGAGACCCGGCTTTCCACGAGCACTTCTTCTTTCCTGTCCAAGAGGAGGATGATCAGAAGCGTCTCTTGGTTACTGTGTGGAACAGGGCCAGCCAGTCCAGACAGAGTGGACTCATTGGCTGCATGAGCTTTGGGGTGAAGTCTCTCCTGACTCCAGACAAGGAGATCAGTGGTTGGTACTACCTCCTAGGGGAGCACCTGGGCCGGACCAAGCACTTGAAGGTGGCCAGGCGGCGACTGCGGCCGCTGAGAGACCCGCTGCTGAGAATGCCAGGAGGTGGGGACACTGAGAATGGGAAGAAACTAAAGATCACCATCCCGAGGGGAAAGGACGGCTTTGGCTTCACCATCTGCTGCGACTCTCCAGTTCGAGTCCAGGCCGTGGATTCCGGGGGTCCGGCGGAACGGGCAGGGCTGCAGCAGCTGGACACGGTGCTGCAGCTGAATGAGAGGCCTGTGGAGCACTGGAAATGTGTGGAGCTGGCCCACGAGATCCGGAGCTGCCCCAGTGAGATCATCCTACTCGTGTGGCGCATGGTCCCCCAGGTCAAGCCAGGACCAGATGGCGGGGTCCTGCGGCGGGCCTCCTGCAAGTCGACACATGACCTCCAGTCACCCCCCAACAAACGGGAGAAGAACTGCACCCATGGGGTCCAGGCACGGCCTGAGCAGCGCCACAGCTGCCACCTGGTATGTGACAGCTCTGATGGGCTGCTGCTCGGCGGCTGGGAGCGCTACACCGAGGTGGCCAAGCGCGGGGGCCAGCACACCCTGCCTGCACTGTCCCGTGCCACTGCCCCCACCGACCCCAACTACATCATCCTGGCCCCGCTGAATCCTGGGAGCCAGCTGCTCCGGCCTGTGTACCAGGAGGATACCATCCCCGAAGAATCAGGGAGTCCCAGTAAAGGGAAGTCCTACACAGGCCTGGGGAAGAAGTCCCGGCTGATGAAGACAGTGCAGACCATGAAGGGCCACGGGAACTACCAAAACTGCCCGGTTGTGAGGCCGCATGCCACGCACTCAAGCTATGGCACCTACGTCACCCTGGCCCCCAAAGTCCTGGTGTTCCCTGTCTTTGTTCAGCCTCTAGATCTCTGTAATCCTGCCCGGACCCTCCTGCTGTCAGAGGAGCTGCTGCTGTATGAAGGGAGGAACAAGGCTGCCGAGGTGACACTGTTTGCCTATTCGGACCTGCTGCTCTTCACCAAGGAGGACGAGCCTGGCCGCTGCGACGTCCTGAGGAACCCCCTCTACCTCCAGAGTGTGAAGCTGCAGGAAGGTTCTTCAGAAGACCTGAAATTCTGCGTGCTCTATCTAGCAGAGAAGGCAGAGTGCTTATTCACTTTGGAAGCGCACTCGCAGGAGCAGAAGAAGAGAGTGTGCTGGTGCCTGTCGGAGAACATCGCCAAGCAGCAACAGCTGGCAGCATCACCCCCGGACAGCAAGATGTTTGAGACGGAGGCAGATGAGAAGAGGGAGATGGCCTTGGAGGAAGGGAAGGGGCCTGGTGCCGAGGATTCCCCACCCAGCAAGGAGCCCTCTCCTGGCCAGGAGCTTCCTCCAGGACAAGACCTTCCACCCAACAAGGACTCCCCTTCTGGGCAGGAACCCGCTCCCAGCCAAGAACCACTGTCCAGCAAAGACTCAGCTACCTCTGAAGGATCCCCTCCAGGCCCAGATGCTCCGCCCAGCAAGGATGTGCCACCATGCCAGGAACCCCCTCCAGCCCAAGACCTCTCACCCTGCCAGGACCTACCTGCTGGTCAAGAACCCCTGCCTCACCAGGACCCTCTACTCACCAAAGACCTCCCTGCCATCCAGGAATCCCCCACCCGGGACCTTCCACCCTGTCAAGATCTGCCTCCTAGCCAGGTCTCCCTGCCAGCCAAGGCCCTTACTGAGGACACCATGAGCTCCGGGGACCTACTAGCAGCTACTGGGGACCCACCTGCGGCCCCCAGGCCAGCCTTCGTGATCCCTGAGGTCCGGCTGGATAGCACCTACAGCCAGAAGGCAGGGGCAGAGCAGGGCTGCTCGGGAGATGAGGAGGATGCAGAAGAGGCCGAGGAGGTGGAGGAGGGGGAGGAAGGGGAGGAGGACGAGGATGAGGACACCAGCGATGACAACTACGGAGAGCGCAGTGAGGCCAAGCGCAGCAGCATGATCGAGACGGGCCAGGGGGCTGAGGGTGGCCTCTCACTGCGTGTGCAGAACTCGCTGCGGCGCCGGACGCACAGCGAGGGCAGCCTGCTGCAGGAGCCCCGAGGGCCCTGCTTTGCCTCCGACACCACCTTGCACTGCTCAGACGGTGAGGGCGCCGCCTCCACCTGGGGCATGCCTTCGCCCAGCACCCTCAAGAAAGAGCTGGGCCGCAATGGTGGCTCCATGCACCACCTTTCCCTCTTCTTCACAGGACACAGGAAGATGAGCGGGGCTGACACCGTTGGGGATGATGACGAAGCCTCCCGGAAGAGAAAGAGCAAAAACCTAGCCAAGGACATGAAGAACAAGCTGGGGATCTTCAGACGGCGGAATGAGTCCCCTGGAGCCCCTCCCGCGGGCAAGGCAGACAAAATGATGAAGTCATTCAAGCCCACCTCAGAGGAAGCCCTCAAGTGGGGCGAGTCCTTGGAGAAGCTGCTGGTTCACAAATACGGGTTAGCAGTGTTCCAAGCCTTCCTTCGCACTGAGTTCAGTGAGGAGAATCTGGAGTTCTGGTTGGCTTGTGAGGACTTCAAGAAGGTCAAGTCACAGTCCAAGATGGCATCCAAGGCCAAGAAGATCTTTGCTGAATACATCGCGATCCAGGCATGCAAGGAGGTCAACCTGGACTCCTACACGCGGGAGCACACCAAGGACAACCTGCAGAGCGTCACGCGGGGCTGCTTCGACCTGGCACAGAAGCGCATCTTCGGGCTCATGGAAAAGGACTCGTACCCTCGCTTTCTCCGTTCTGACCTCTACCTGGACCTTATTAACCAGAAGAAGATGAGTCCCCCGCTTTAG

**RGS3 overexpression Sequence (RGS3-3Flag)**

GGATCCGCAGAGTGCTTATTCACTTTGTTCAAGAGACAAAGTGAATAAGCACTCTGCTTTTTTGAATTCGAATTCGCCACCATGGAGTGGCTAAGCCCTGATATCGCTCTGCCCAGAAGAGATGAGTGGACTCAAACTTCTCCAGCCAGGAAGAGGATCACGCATGCCAAAGTCCAGGGTGCAGGTCAGCTGAGGCTGTCCATTGATGCCCAGGACCGGGTTCTGCTGCTTCACATTATAGAAGGTAAAGGCCTGATCAGCAAACAGCCTGGCACCTGTGATCCGTATGTGAAGATTTCTTTGATCCCTGAAGATAGTAGACTACGCCACCAGAAGACGCAGACCGTTCCAGACTGCAGAGACCCGGCTTTCCACGAGCACTTCTTCTTTCCTGTCCAAGAGGAGGATGATCAGAAGCGTCTCTTGGTTACTGTGTGGAACAGGGCCAGCCAGTCCAGACAGAGTGGACTCATTGGCTGCATGAGCTTTGGGGTGAAGTCTCTCCTGACTCCAGACAAGGAGATCAGTGGTTGGTACTACCTCCTAGGGGAGCACCTGGGCCGGACCAAGCACTTGAAGGTGGCCAGGCGGCGACTGCGGCCGCTGAGAGACCCGCTGCTGAGAATGCCAGGAGGTGGGGACACTGAGAATGGGAAGAAACTAAAGATCACCATCCCGAGGGGAAAGGACGGCTTTGGCTTCACCATCTGCTGCGACTCTCCAGTTCGAGTCCAGGCCGTGGATTCCGGGGGTCCGGCGGAACGGGCAGGGCTGCAGCAGCTGGACACGGTGCTGCAGCTGAATGAGAGGCCTGTGGAGCACTGGAAATGTGTGGAGCTGGCCCACGAGATCCGGAGCTGCCCCAGTGAGATCATCCTACTCGTGTGGCGCATGGTCCCCCAGGTCAAGCCAGGACCAGATGGCGGGGTCCTGCGGCGGGCCTCCTGCAAGTCGACACATGACCTCCAGTCACCCCCCAACAAACGGGAGAAGAACTGCACCCATGGGGTCCAGGCACGGCCTGAGCAGCGCCACAGCTGCCACCTGGTATGTGACAGCTCTGATGGGCTGCTGCTCGGCGGCTGGGAGCGCTACACCGAGGTGGCCAAGCGCGGGGGCCAGCACACCCTGCCTGCACTGTCCCGTGCCACTGCCCCCACCGACCCCAACTACATCATCCTGGCCCCGCTGAATCCTGGGAGCCAGCTGCTCCGGCCTGTGTACCAGGAGGATACCATCCCCGAAGAATCAGGGAGTCCCAGTAAAGGGAAGTCCTACACAGGCCTGGGGAAGAAGTCCCGGCTGATGAAGACAGTGCAGACCATGAAGGGCCACGGGAACTACCAAAACTGCCCGGTTGTGAGGCCGCATGCCACGCACTCAAGCTATGGCACCTACGTCACCCTGGCCCCCAAAGTCCTGGTGTTCCCTGTCTTTGTTCAGCCTCTAGATCTCTGTAATCCTGCCCGGACCCTCCTGCTGTCAGAGGAGCTGCTGCTGTATGAAGGGAGGAACAAGGCTGCCGAGGTGACACTGTTTGCCTATTCGGACCTGCTGCTCTTCACCAAGGAGGACGAGCCTGGCCGCTGCGACGTCCTGAGGAACCCCCTCTACCTCCAGAGTGTGAAGCTGCAGGAAGGTTCTTCAGAAGACCTGAAATTCTGCGTGCTCTATCTAGCAGAGAAGGCAGAGTGCTTATTCACTTTGGAAGCGCACTCGCAGGAGCAGAAGAAGAGAGTGTGCTGGTGCCTGTCGGAGAACATCGCCAAGCAGCAACAGCTGGCAGCATCACCCCCGGACAGCAAGATGTTTGAGACGGAGGCAGATGAGAAGAGGGAGATGGCCTTGGAGGAAGGGAAGGGGCCTGGTGCCGAGGATTCCCCACCCAGCAAGGAGCCCTCTCCTGGCCAGGAGCTTCCTCCAGGACAAGACCTTCCACCCAACAAGGACTCCCCTTCTGGGCAGGAACCCGCTCCCAGCCAAGAACCACTGTCCAGCAAAGACTCAGCTACCTCTGAAGGATCCCCTCCAGGCCCAGATGCTCCGCCCAGCAAGGATGTGCCACCATGCCAGGAACCCCCTCCAGCCCAAGACCTCTCACCCTGCCAGGACCTACCTGCTGGTCAAGAACCCCTGCCTCACCAGGACCCTCTACTCACCAAAGACCTCCCTGCCATCCAGGAATCCCCCACCCGGGACCTTCCACCCTGTCAAGATCTGCCTCCTAGCCAGGTCTCCCTGCCAGCCAAGGCCCTTACTGAGGACACCATGAGCTCCGGGGACCTACTAGCAGCTACTGGGGACCCACCTGCGGCCCCCAGGCCAGCCTTCGTGATCCCTGAGGTCCGGCTGGATAGCACCTACAGCCAGAAGGCAGGGGCAGAGCAGGGCTGCTCGGGAGATGAGGAGGATGCAGAAGAGGCCGAGGAGGTGGAGGAGGGGGAGGAAGGGGAGGAGGACGAGGATGAGGACACCAGCGATGACAACTACGGAGAGCGCAGTGAGGCCAAGCGCAGCAGCATGATCGAGACGGGCCAGGGGGCTGAGGGTGGCCTCTCACTGCGTGTGCAGAACTCGCTGCGGCGCCGGACGCACAGCGAGGGCAGCCTGCTGCAGGAGCCCCGAGGGCCCTGCTTTGCCTCCGACACCACCTTGCACTGCTCAGACGGTGAGGGCGCCGCCTCCACCTGGGGCATGCCTTCGCCCAGCACCCTCAAGAAAGAGCTGGGCCGCAATGGTGGCTCCATGCACCACCTTTCCCTCTTCTTCACAGGACACAGGAAGATGAGCGGGGCTGACACCGTTGGGGATGATGACGAAGCCTCCCGGAAGAGAAAGAGCAAAAACCTAGCCAAGGACATGAAGAACAAGCTGGGGATCTTCAGACGGCGGAATGAGTCCCCTGGAGCCCCTCCCGCGGGCAAGGCAGACAAAATGATGAAGTCATTCAAGCCCACCTCAGAGGAAGCCCTCAAGTGGGGCGAGTCCTTGGAGAAGCTGCTGGTTCACAAATACGGGTTAGCAGTGTTCCAAGCCTTCCTTCGCACTGAGTTCAGTGAGGAGAATCTGGAGTTCTGGTTGGCTTGTGAGGACTTCAAGAAGGTCAAGTCACAGTCCAAGATGGCATCCAAGGCCAAGAAGATCTTTGCTGAATACATCGCGATCCAGGCATGCAAGGAGGTCAACCTGGACTCCTACACGCGGGAGCACACCAAGGACAACCTGCAGAGCGTCACGCGGGGCTGCTTCGACCTGGCACAGAAGCGCATCTTCGGGCTCATGGAAAAGGACTCGTACCCTCGCTTTCTCCGTTCTGACCTCTACCTGGACCTTATTAACCAGAAGAAGATGAGTCCCCCGCTTGATTACAAGGATGACGACGATAAGGACTATAAGGACGATGATGACAAGGACTACAAAGATGATGACGATAAATAGGGATCC

Lentiviral vectors containing RGS3 short hairpin RNA (shRNA) and RGS3 overexpression constructs synthesized by General Biosystems (Anhui, China) are shown above. Control groups were infected with an empty vector.
